# X-ray computed tomography datasets for forensic analysis of vertebrate fossils

**DOI:** 10.1038/sdata.2016.40

**Published:** 2016-06-07

**Authors:** Timothy B. Rowe, Zhe-Xi Luo, Richard A. Ketcham, Jessica A. Maisano, Matthew W. Colbert

**Affiliations:** 1 High-Resolution X-ray CT Facility, Department of Geological Sciences, The University of Texas, Austin, Texas 78712, USA; 2 Department of Anatomy, University of Chicago, Chicago, Illinois 60637, USA

**Keywords:** Research data, Palaeontology, X-ray tomography

## Abstract

We describe X-ray computed tomography (CT) datasets from three specimens recovered from Early Cretaceous lakebeds of China that illustrate the forensic interpretation of CT imagery for paleontology. Fossil vertebrates from thinly bedded sediments often shatter upon discovery and are commonly repaired as amalgamated mosaics grouted to a solid backing slab of rock or plaster. Such methods are prone to inadvertent error and willful forgery, and once required potentially destructive methods to identify mistakes in reconstruction. CT is an efficient, nondestructive alternative that can disclose many clues about how a specimen was handled and repaired. These annotated datasets illustrate the power of CT in documenting specimen integrity and are intended as a reference in applying CT more broadly to evaluating the authenticity of comparable fossils.

## Background & Summary

**Figure 1:**
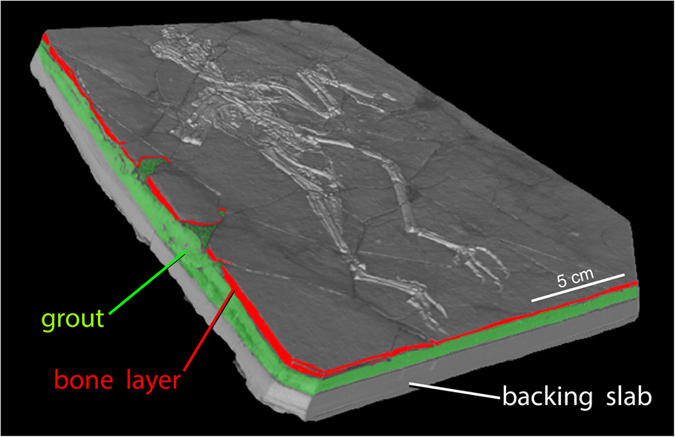
The *Confuciusornis* amalgamation, in oblique view. Volumetric rendering from CT data (Data Citation 1) showing its 3-layer man-made stratigraphy. The top layer is only 2–8 mm thick and was shattered during excavation. It was mosaicked together using a ceramic grout on a backing slab that provided structural integrity. Edges of the top bone-bearing layer are colored red; the middle layer consisting of grout is green; and the backing slab is gray.

Vertebrate fossils are often broken upon discovery and during excavation, and must be reassembled for research and display. This is especially true for specimens preserved on bedding planes in thinly laminated sediments, which can completely shatter. Simply gluing together the broken edges rarely yields a structurally sound specimen because the edges are too thin. Repair commonly involves mosaicking the broken pieces together, using plaster or grout, onto a stable ‘backing-slab’ that provides structural integrity. The product is a three-layered object whose ‘stratigraphy’ is man-made (Fig. 1).

A two-century-long history indicates that this type of reassembly is prone to inadvertent error and willful forgery^1–7^. Surface layers often include extraneous pieces to fill voids around the restored skeleton. It is not uncommon to discover that bones from different specimens, even taxa, were combined to create the impression of a single ‘complete’ skeleton. Surfaces are often painted and textured to disguise repairs and give visual continuity to the whole. For larger specimens, it was once common practice to build a structural wooden frame around the reconstructed slab that obscured its ‘stratigraphy.’ Painted or grouted edges also hide evidence of reconstruction and obscure interpretation.

Once reassembled, these are generally considered to be ‘specimens’ in the conventional sense. Here, we emphasize that they are more aptly viewed as ‘*amalgamations’* since they often comingle associated bone-bearing pieces with extraneous rocks and consolidating materials. Unrecognized extraneous elements can lead to cascading errors in scientific analyses.

The problem grew significantly in the last two decades as fossiliferous deposits in China began to yield prodigious quantities of fossils, many of which were quite complete and well-preserved^8–24^. However, many of the new specimens, including holotypes, were reportedly excavated and reassembled autonomously by local farmers who sold them to private collectors, researchers, and museums^25–29^. In such cases, a scientific ‘chain-of-possession’ is difficult to establish^25–34^. Although strictly illegal^35^, commercialization of Chinese fossils and a quasi-free-market of fossil trade are widespread owing to weak law enforcement^36^. Commercialization also provides financial incentives to cosmetically enhance imperfect fossils, causing scientific damage^26–29^.

Some prominent scientists claim that ‘Normally we know right away if a fossil is a fake…’^34^. Indeed, a published fossil skull^37^ was recently exposed as a forgery using conventional techniques^38^ and the publication was retracted^39^. But conventional preparation is limited by its invasive nature, and for logistical and technical reasons other scientists concede that ‘Authentication is not easy’^34^.

With this widely recognized problem, we endorse the recommendation that authentication of fossils not directly collected by scientists should be a required research protocol^36^. Chinese authorities have taken legislative steps to prevent illegal trafficking in fossils^35,40,41^ but a black market has existed for years^26–29^ and concerns are voiced that ‘The fake fossil problem has become very, very serious’^34^. These problems, highlighted by China, are global in nature^1–4,7^.

Computed tomography (CT) has been used for 30 years to nondestructively inspect the entire 3D volumes of fossils^42–48^. CT can reveal many features otherwise invisible such as endocasts of the brain^49–53^ and inner ear^54,55^. Several amalgamations and forgeries have passed through the University of Texas High-Resolution X-ray Computed Tomography Facility (UTCT) in its 19 years of operation. Here, we describe protocols that we developed to authenticate fossils by validating the associations between pieces and to identify extraneous elements^6^.

More general application of CT to forensic problems in paleontology is hindered by the lack of exemplar datasets and published assay procedures. We describe three datasets from Early Cretaceous lacustrine deposits of Liaoning, P. R. China that illustrate the use of CT in forensic analysis. The first is a specimen of the primitive bird *Confuciusornis*^56^ that was shattered and reconstructed with relatively minor errors. The second is the so-called ‘Archaeoraptor’^57^, a chimaera of multiple taxa^6^. The third is a nearly perfect specimen of the primitive mammal *Jeholodens*^58^. Our intention is that these datasets may assist others in properly interpreting CT imagery to evaluate the integrity of individual specimens, and to extend the application of CT in authenticating fossils.

## Methods

### Material

#### *Confuciusornis lacustris*

**Figure 2:**
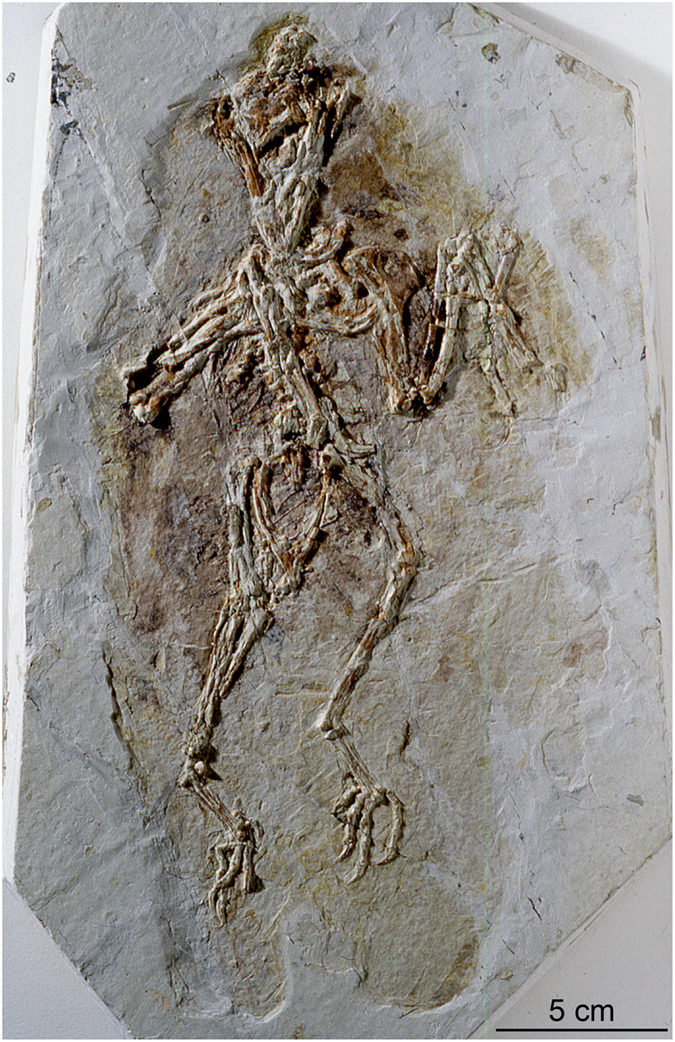
Photograph of the *Confuciusornis* amalgamation as it was presented for CT scanning in 1998, taken with 35 mm color slide film (Photography by Joe Jaworowski).

**Figure 3:**
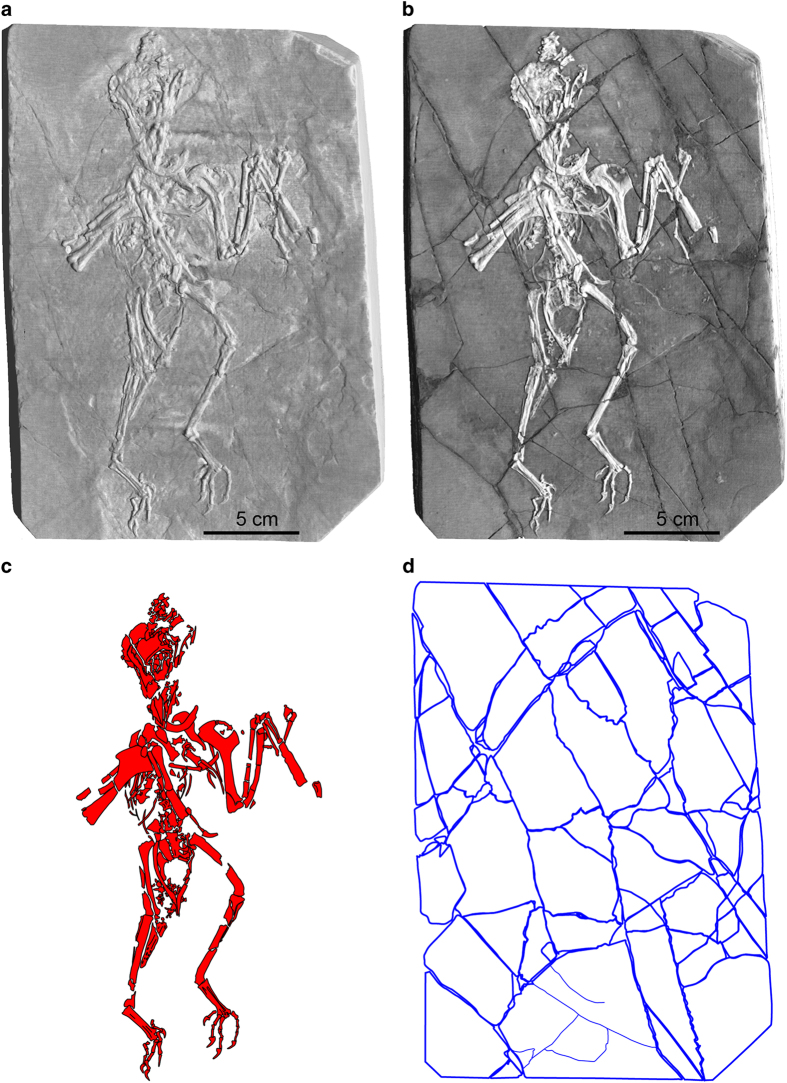
A 3-D surface model of the *Confuciusornis* amalgamation (**a**), compared to a 3-D volumetric rendering (**b**) that shows more vividly its extensive fracture pattern. Both were generated from the same high-resolution X-ray CT dataset (Data Citation 1). The skeleton (**c**) was digitally filtered from the rest of the amalgamation. The shatter-fracture pattern of the bone-bearing top layer (**d**) was mapped from the volumetric rendering and from cross-sections.

**Figure 4:**
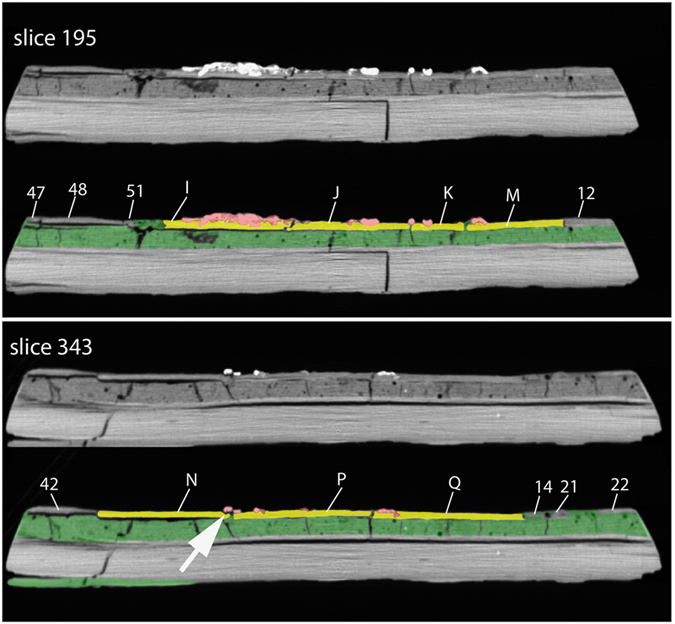
*Confuciusornis* amalgamation, showing slices 195 and 343 (see Fig. 5 for slice locations) in original gray scale (above), and color coded (below). Contrast was adjusted and colors were added in Adobe *Photoshop*. Letters refer to the validated bone-bearing pieces and numbers refer to shims labeled in Fig. 5. In slice 343, the arrow points to a thin, black separation between a shard of the right femur (red) and the rest of the slab (yellow) filled with a low density consolidant, indicating that this piece was glued back onto the slab.

**Figure 5:**
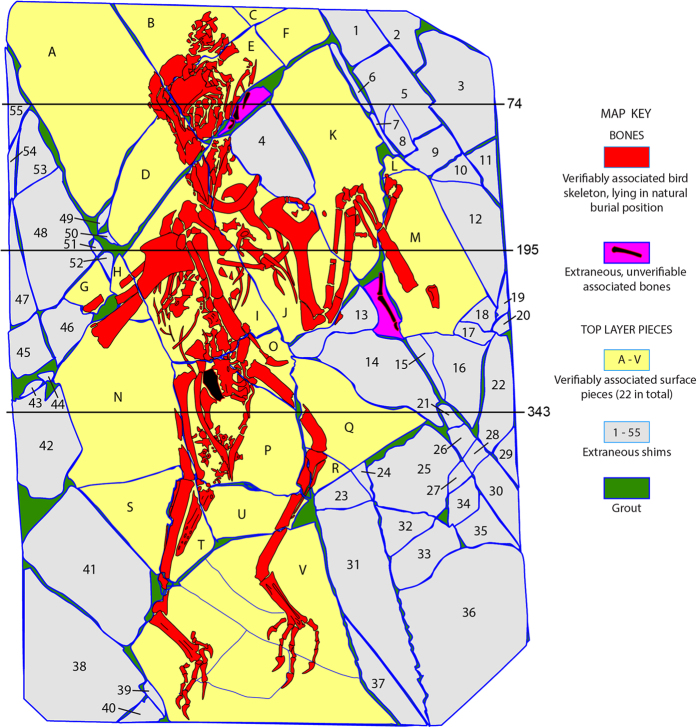
Map of the *Confuciusornis* amalgamation derived from high-resolution X-ray CT data (Data Citation 1). The skeleton is in red and its associated shale is yellow. Extraneous bone-bearing pieces are magenta, construction shims are gray, and grout is green. The numbered lines indicate the positions of slice planes for original CT data labeled in Figs 4 and 6.

**Figure 6:**
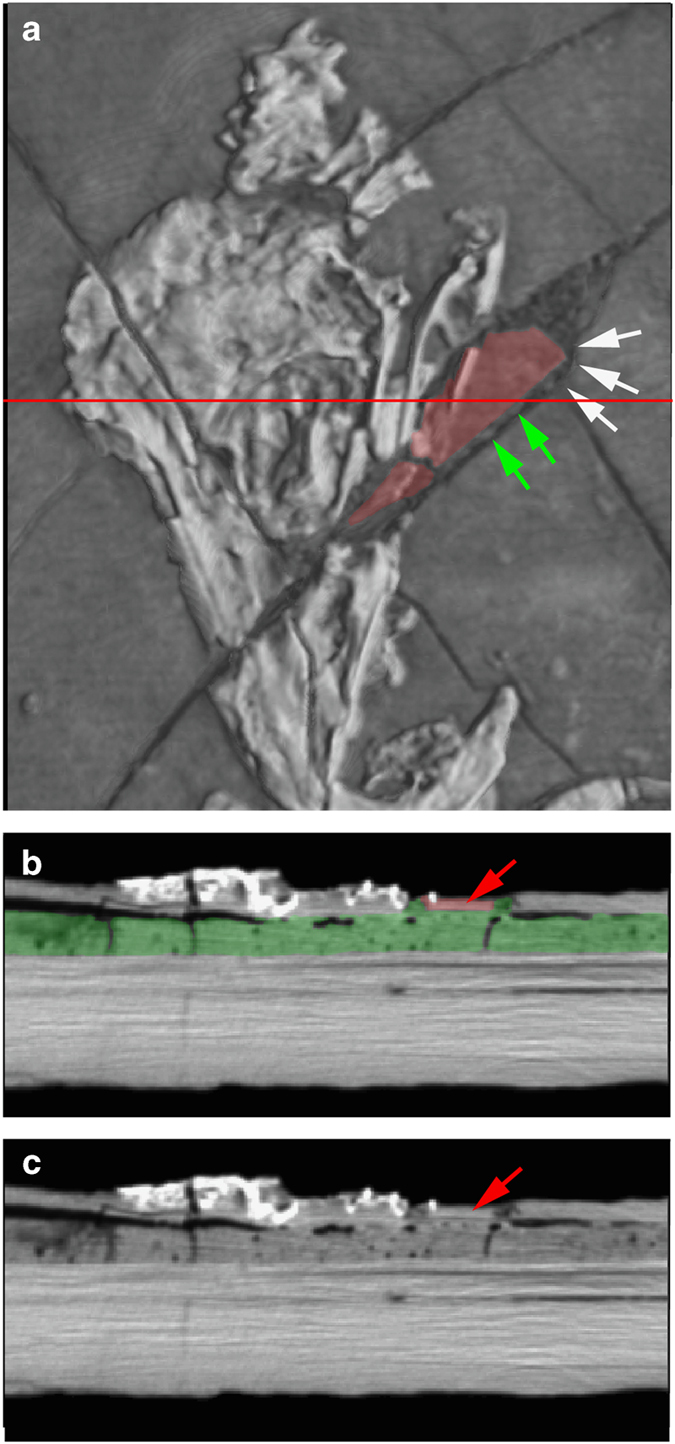
3-D volume rendering of the *Confuciusornis* skull, showing two mismatched admixed fragments in map view (**a**). The horizontal red line indicates the position of slice 74, which is shown as a colored cross section (**b**), and cross section without color (**c**). Color was added in Adobe *Photoshop*, to highlight the mismatched pieces. White arrows (**a**) indicate a mismatched rounded edge against which the squared corner of the extraneous red piece was fitted. Green arrows indicate ‘strings’ of grout used to hold the extraneous piece in place. Red arrows (**b**,**c**) point to the extraneous piece in cross section; note how thin it is compared to the pieces on either side, and that it is separated from adjacent bones by grout.

(Figs 1,2,3,4,5,6; see Table 1 for scanning parameters; Table 2 for data output; Table 3 for movies; see also Supplementary Figures; Data Citation 1; additional information is available at: http://digimorph.org/specimens/Confuciusornis_sp/skeleton/). This unnumbered specimen was provided to us for scanning in 1998 by Mr Guan Jian of the Beijing Museum of Natural History, as an early test of whether specimens from the newly discovered Liaoning basin were amenable to CT scanning^59^. It was reportedly collected from the lower Yixian Formation, but its precise locality within the Liaoning basin is unknown and it came to us with no other documentation. It is now housed in the Institute for Vertebrate Paleontology and Paleoanthropology in Beijing.

#### ‘Archaeoraptor liaoningensis’

**Figure 7:**
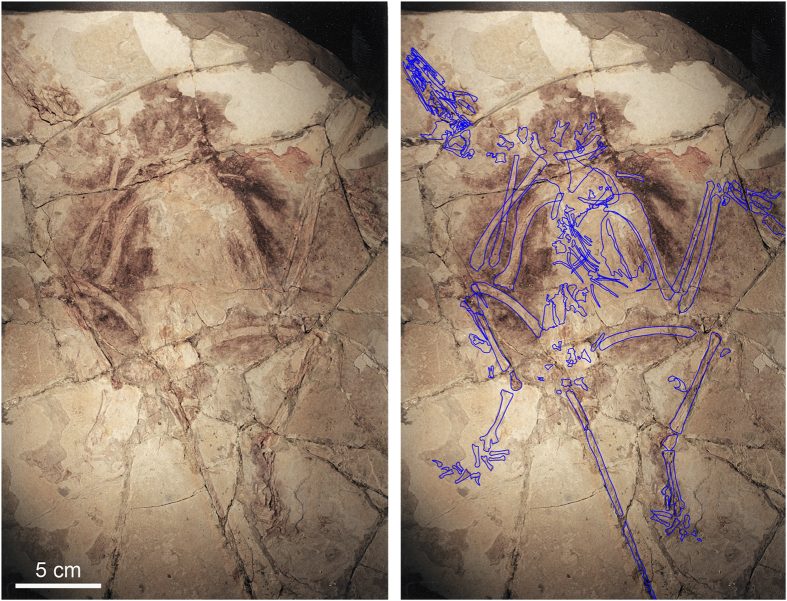
Photograph of the ‘Archaeoraptor’ amalgamation taken as it was presented for CT scanning at UTCT in August, 1999. A series of professional large-format photographs taken in visible and ultraviolet light is available for viewing at http://digimorph.org/specimens/Archaeoraptor_forgery/.

**Figure 8:**
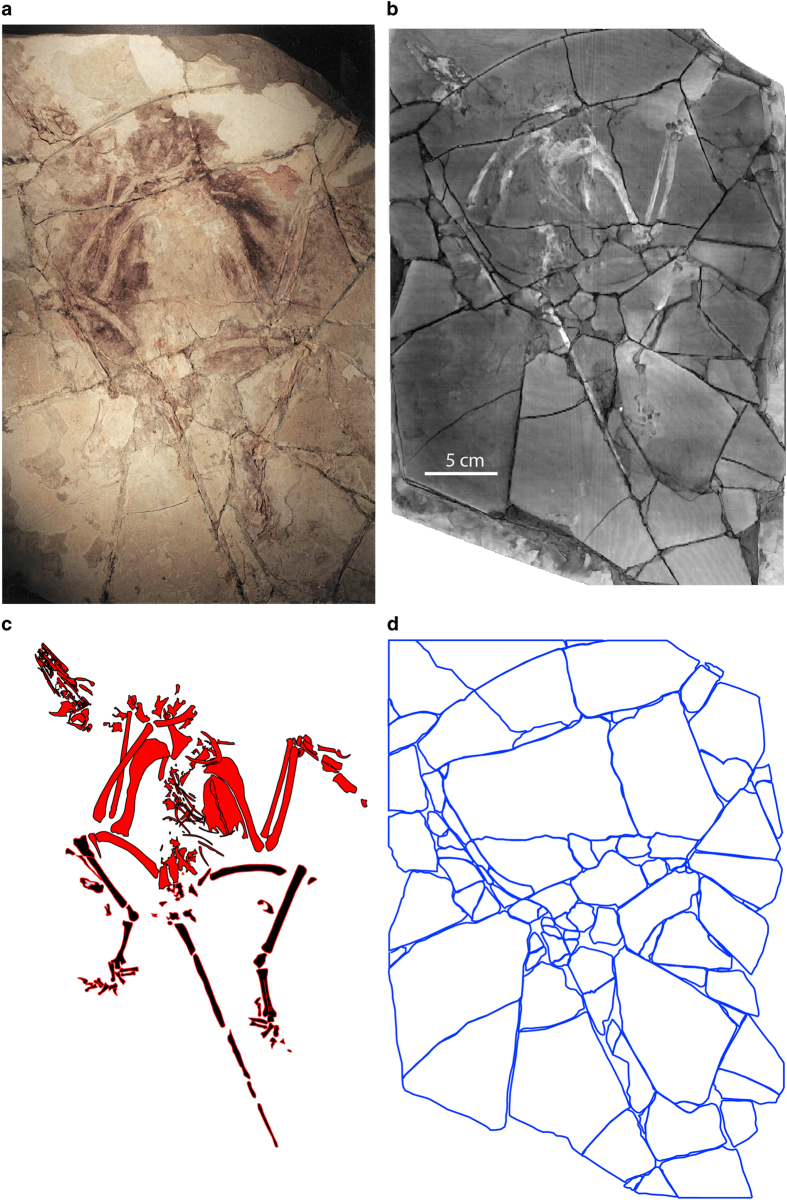
‘Archaeoraptor’ amalgamation. Photo (**a**), volumetric reconstruction (**b**), silhouette of skeleton (red) and associated bones (black) (**c**), and fracture map of the top bone-bearing layer (**d**) (Data Citation 1).

**Figure 9:**
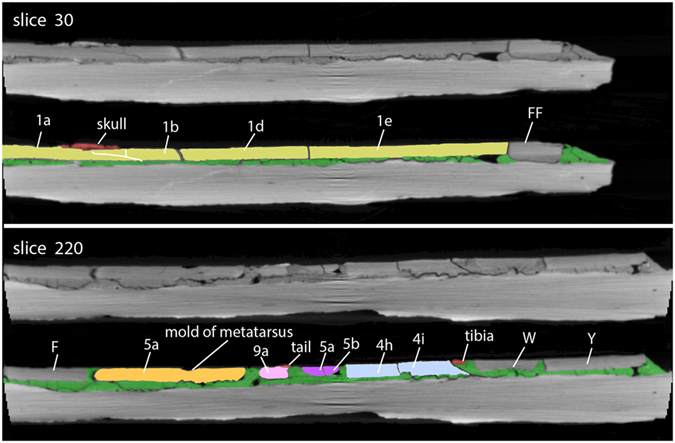
‘Archaeoraptor’ amalgamation, showing slices 30 and 220 in original grayscale (above), and color coded (below). Contrast was increased and colors were added in Adobe *Photoshop*. Letters and numbers are keyed to Fig. 10.

**Figure 10:**
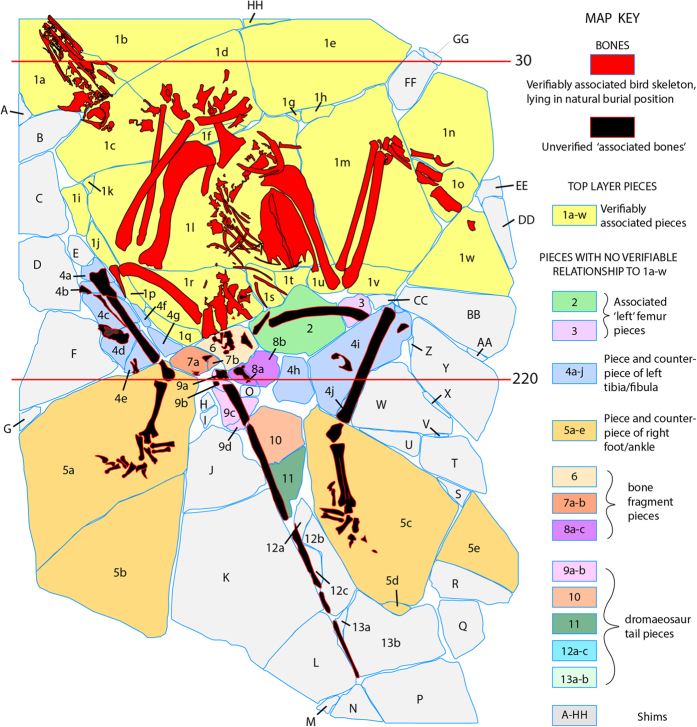
Map of the ‘Archaeoraptor’ amalgamation, as it was presented for CT scanning at the University of Texas High-Resolution X-ray CT facility on July 29, 1999, with a key to its various parts. The numbered lines indicate slice planes for original CT data labeled in Fig. 9.

**Figure 11:**
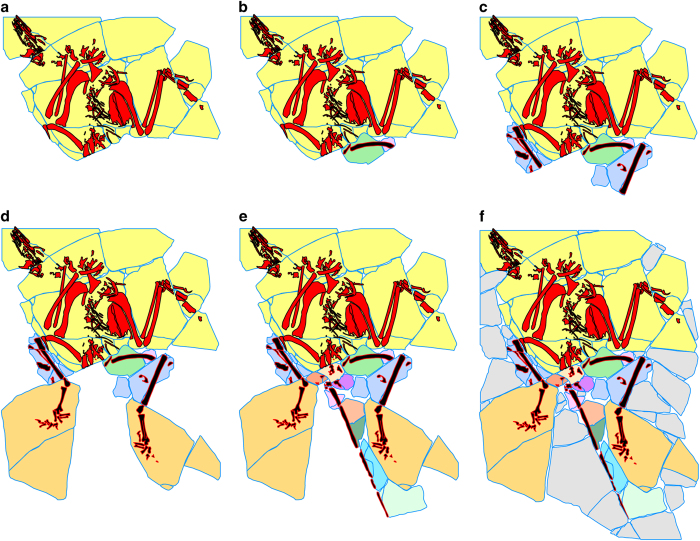
The probable sequence of assembly of the top layer of the ‘Archaeoraptor’ amalgamation, starting with (**a**) the partial *Yanornis* skeleton, to which a split femur (**b**) was added, followed by the tibiae (**c**), the split (part and counter-part) foot (**d**), the tail (**e**), and finally the shims (**f**).

The ‘Archaeoraptor’ amalgamation (Figs 7,8,9,10,11; see Table 1 for scanning parameters; Table 2 for data output; Table 3 for movies; see also Supplementary Figures; Data Citation 1; additional information is available at: http://digimorph.org/specimens/Archaeoraptor_forgery/) was reportedly collected from the Early Cretaceous Jiufotang Formation of Liaoning, but no documentation accompanied it^57^. It was provided for scanning by Steven and Sylvia Czerkas of the Dinosaur Museum, Blanding, Utah, under a grant from the National Geographic Society to Dr Philip Currie of the Royal Tyrrell Museum of Palaeontology, Drumheller, Alberta. The specimen was later disclosed to have been smuggled from China and sold for $80,000 in the US^18–21,24–28^, as a purported missing link between birds and more primitive theropod dinosaurs^57^. It was repatriated to the Institute for Vertebrate Paleontology and Paleoanthropology in Beijing in 2000.

A series of 22 large-format photographs of the specimen’s surface was taken by Mr. Lou Mazzatenta for National Geographic Creative in 1999. They were taken using visible and ultraviolet light, and can be viewed at www.DigiMorph.org/specimens/Archaeoraptor_forgery. These photos were taken after we CT scanned the amalgamation, and we note that one of its extraneous pieces (Piece F, below) was subsequently removed and is absent in the photographs.

#### *Jeholodens jenkinsi*

**Figure 12:**
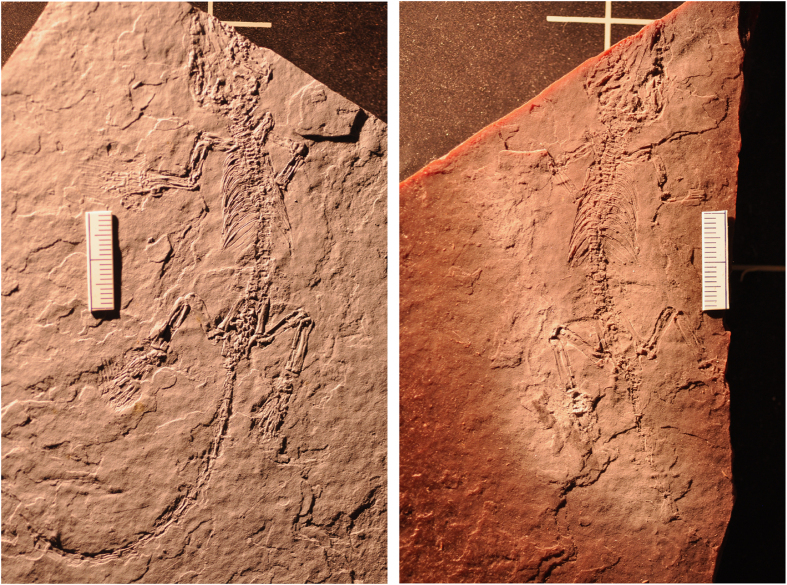
Photographs of part- and counter-part of *Jeholodens jenkinsi* as they were split upon discovery. Only the main part (left) was CT scanned (Data Citation 1).

**Figure 13:**
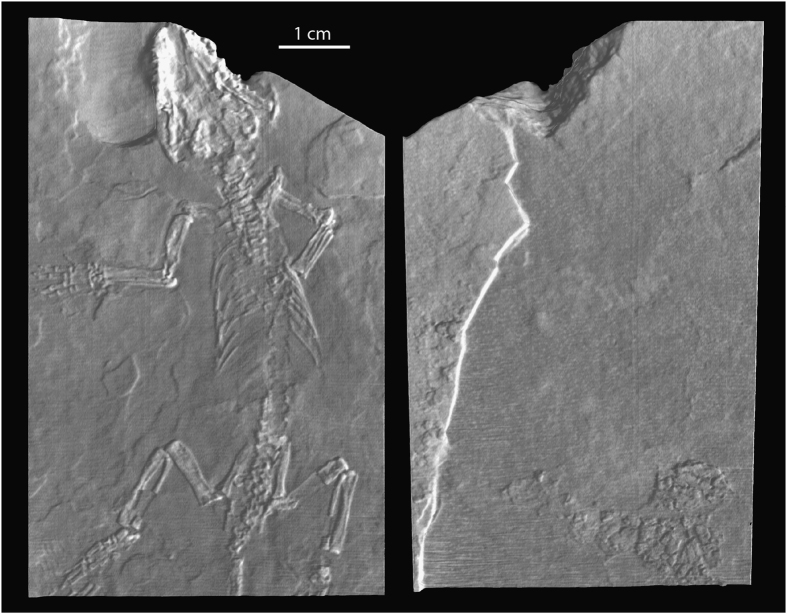
Volume rendering from CT data (Data Citation 1) of the *Jeholodens* specimen, showing the top (left) and bottom (right) of the main slab. This small slab is virtually devoid of fractures.

**Figure 14:**
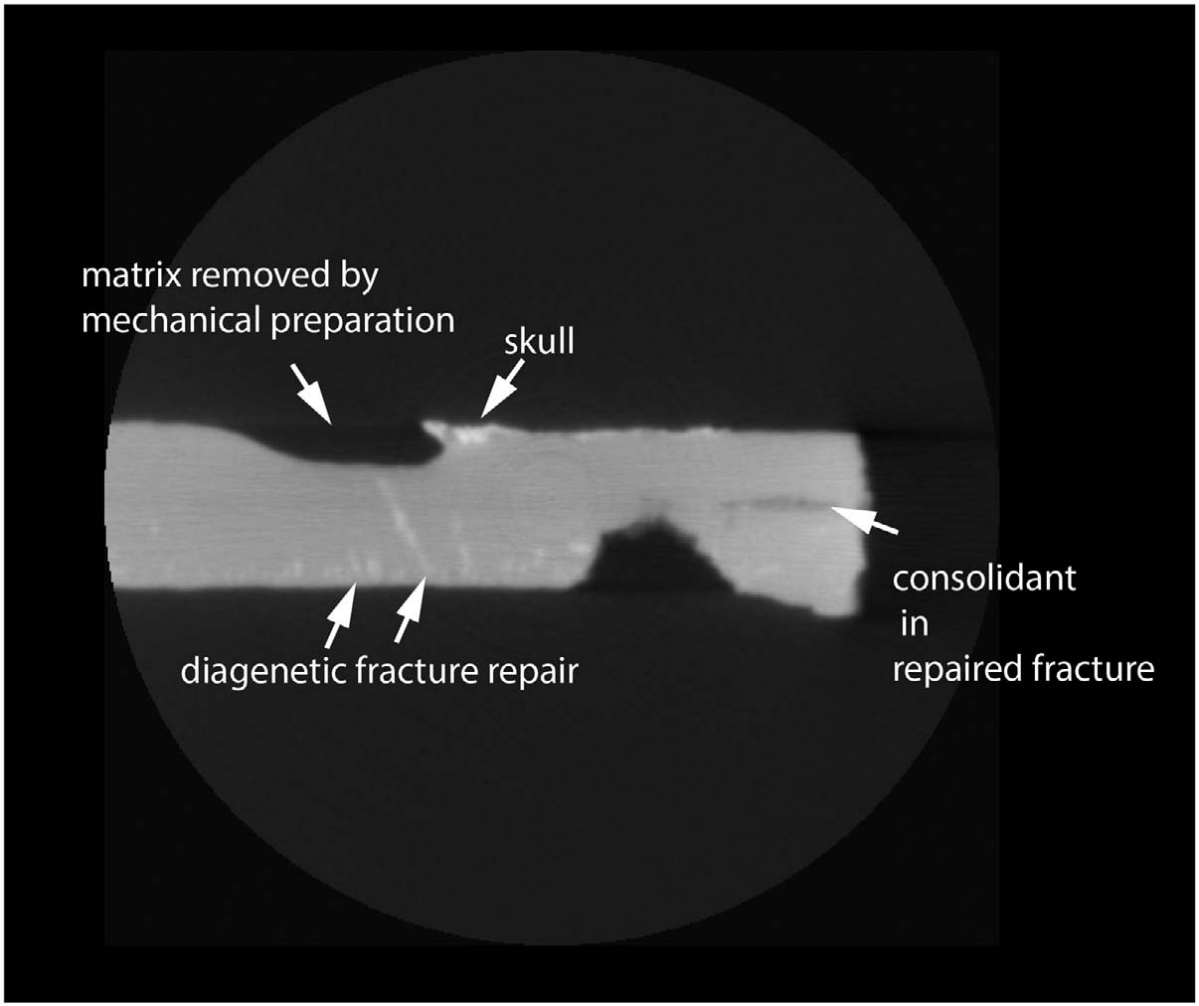
*Jeholodens*. Sample XY section (slice 59).

National Geological Museum of China, holotype, specimen number GMW 2139a (Figs 12,13,14; see Table 1 for scanning parameters; Table 2 for data output; Table 3 for movies; see also Supplementary Figures; Data Citation 1; additional information is available at: http://digimorph.org/specimens/Jeholodens_jenkinsi/)^58^. The only known specimen of *Jeholodens jenkinsi* was made available for scanning by Dr Zhe-Xi Luo, then Curator at the Carnegie Museum of Natural History, Pittsburgh, Pennsylvania. It was recovered by an unknown collector in the Early Cretaceous Yixian Formation, reportedly from the Sihetun site, Liaoning Province, P.R. China, and obtained by the National Geological Museum of China.

### Scanning Protocols and Parameters

All three specimens were scanned at UTCT using an ACTIS scanner manufactured in 1997 (with routine subsequent upgrades) by BioImaging Research, of Lincolnshire, Illinois. The scanner used to generate these datasets is now decommissioned. For the sake of procedural clarity, its technical specifications and the parameters used to scan each specimen are detailed here. The evolution of CT instrumentation and protocols has been thoroughly described elsewhere^43–47,60^.

For all three specimens, we used our high-energy subsystem with scanning parameters recorded in Table 1. At the time, this system employed a Pantak 420-kV tungsten X-ray source, a rotating turntable that accommodated samples up to 50 kg in weight, and either of two possible high-energy detectors. One detector (P250D), a 512-channel cadmium tungstate solid-state linear array, provided the greatest sensitivity among our detectors because of its high absorption efficiency. Its vertical aperture (slice thickness) could range from 5 mm down to 0.25 mm, with a horizontal channel pitch of 0.31 mm. The Radiographic Line Scanner (RLS) detector, a 2048-channel gadolinium oxysulfide linear array, provided lower sensitivity than the P250D but higher in-plane spatial resolution, with a channel pitch of 0.025 mm. It could be used either in a high-resolution mode, with a vertical aperture of 0.25 mm, or (by sacrificing some sensitivity) using a vertical aperture that could vary from 0.5 to 5 mm.

*Jeholodens* and the Archaeoraptor amalgamation were mounted in a Plexiglas cylinder using florist’s foam to prop them upright with the longest axis vertical. CT slices were taken perpendicular to the slab face. The *Confuciusornis* amalgamation was stabilized inside a Plexiglas tube by leaning it at an angle of about 6° against the cylinder walls. In all three specimens the long axis of the slab corresponds roughly to the vertebral axis and longest dimension of the skeleton in its death posture.

#### *Confuciusornis lacustris*

All scanning was done by Richard Ketcham and Timothy Rowe on July 16–17, 1998. The *Confuciusornis* amalgamation was scanned in 0.5-mm-thick slices, with an inter-slice spacing (i.e., distance between slice centers) of 0.45 mm. This is achieved by overlapping the scan planes by 0.05 mm, effectively oversampling the data. This generated 618 consecutive slices. Each reconstructed slice was 1024×1024 pixels with a field of view of 220 mm, resulting in an inter-pixel spacing (in-plane resolution) of 0.215 mm. See Table 1 for additional scan parameters and Table 2 for data outputs (Data Citation 1).

Because the specimen was leaning at a ~6° angle for stability, reslicings in the YZ and XZ planes are not perfectly orthogonal to the slab face The specimen was gently wedged in place with a piece of corrugated cardboard that is visible in some of the slices.

#### ‘Archaeoraptor’

All scanning of the Archaeoraptor amalgamation was done by Richard Ketcham on July 29, 1999—August 4, 1999. The skeleton was scanned from the nose to the end of the tail, generating a total of 422 consecutive 1-mm-thick slices taken at an inter-slice spacing of 0.9 mm. The split bones yielded lower CT contrast with surrounding rock than the *Confuciusornis* specimen, and the greater cross-sectional dimension led to increased streaking and beam-hardening artifacts. As a result, it was necessary to acquire comparatively thick slices. Still, the image quality was not as good as for *Confuciusornis*, and the poorer quality of the specimen is evident in both the CT slices and 3D volumetric reconstructions of the slab. Each reconstructed slice was 1024×1024 pixels with a field of view of 270 mm, resulting in an inter-pixel spacing of 0.264 mm; the slices were subsequently cropped to omit empty space to make storage and rendering more efficient. See Table 1 for additional scan parameters and Table 2 for data outputs (Data Citation 1).

#### *Jeholodens jenkinsi*

Scanning was performed by Richard Ketcham and Matthew Colbert on April 23, 1999. It was scanned from the tip of the skull to the back of the ankles, omitting much of the tail. The anterior 80 mm of the slab were scanned with a slice thickness of 0.25 mm and an inter-slice spacing of 0.20 mm, for a total of 400 slices. Each reconstructed slice was 1024×1024 pixels with a field of view of 50 mm, resulting in an inter-pixel spacing of 0.049 mm. See Table 1 for additional scan parameters and Table 2 for data outputs (Data Citation 1).

We scanned only the main skeleton-bearing part. The small slab containing the skeleton is composed of fine-grained shale and measures approximately 10 cm in length and 1 cm thick. The slab is triangular, with the nose of the skull at the apex, and the postcranium is distributed over an area of increasing width towards the base of the triangle. The triangular geometry is significant because the X-ray beam had to penetrate an increasingly wide expanse of shale towards the base of the triangle. As a result, data quality diminished from front to back of the slab (see below).

### Image Processing

#### Overview

The original CT scans (Data Citation 1) were produced using a 12-bit linear detector that acquired a volume with non-cubic voxels. In other words, the inter-pixel spacing (X and Y) is smaller than than the inter-slice spacing (Z). The 12-bit data were exported in 16-bit format, which makes them appear ‘black’ when opened in many image viewers. To see this imagery, one must adjust image ‘levels’ in most image viewers, either by changing the color table mapping the data values in the file to the displayed gray levels, or by rescaling the data values themselves. Opening the files in *ImageJ* (National Institutes of Health) automatically sets viewable grayscale levels (see Usage Notes). Image stacks can also be imported into *ImageJ* as ‘virtual stacks’ if the user does not have enough RAM to load the image stack directly.

To make these data more easily accessible to those who might have difficulty using them in their original format, we included modified versions in which the grayscale values have been rescaled to better ‘fill’ the 16-bit grayscale space. We also include a version in which the data have been resampled to render the voxels cubic or isometric (Table 2). Many software packages assume that voxels are cubic, thus these resampled datasets may be easier to load under default settings. No discernible artifacts were introduced by the resampling process.

The original scan orientation of these fossils on slabs was chosen to maximize the data quality—which may not yield the most informative images of the specimens. Accordingly, orthogonal reslicings of the data are also provided (YZ and XZ; Data Citation 1; the YZ reslicings provide the best views of the specimen). Finally, we have included 8-bit versions of the rescaled and resliced data for those who do not possess the computing capabilities to process the 16-bit data.

#### Image Processing Methods

Grayscale levels for the ‘16bit_XY’ data were adjusted from the ‘16bit_original’ data using *ImageJ*. To change the grayscale levels, the original data were imported into *ImageJ* as an image sequence, and the grayscale levels were adjusted using the following commands: **Process>Math>Multiply**…**Value: 16**. The modified data were then saved as a TIFF image sequence. We note that this method is a form of ‘windowing’ the data, and that in effect it multiplies a grayscale level from 2^12^ by 2^4^ to make it a fraction of 2^16^.

To generate the ‘8bit_XY’, the ‘16bit_XY’ data were reformatted to 8-bit mode using *ImageJ*: **Image>Type>8 bit**, and saved as a TIFF image sequence. Changing the data from 16-bit to 8-bit format reduces the detail of grayscale information in the data, and correspondingly reduces the file size by 50%, which may make the data accessible for those lacking the necessary computer resources to handle the 16-bit data.

The ‘16bit_resampled’ data were generated by importing the ‘16bit_XY’ data into *Avizo* 8.1 (FEI Company) and resampling them to make the voxels cubic. This was accomplished using the following procedure: **Compute>Volume Operations>Resample>Create**. In the ‘Properties’ for the ‘Resample’ icon the voxel sizes for X, Y, and Z were changed to correspond to the original XY (inter-pixel) resolution (0.215 mm for *Confuciusornis*; 0.264 mm for ‘Archaeoraptor’; and 0.049 mm for *Jeholodens*). The resulting resampled data were then saved as a stack of 2-D TIFF images, and subsequently cropped in *ImageJ* to reduce file size.

The ‘16bitresliceXZ’ and ‘16bitresliceYZ’ comprise orthogonal reslicings of the ‘16bit_resampled’ data set. The reslicings were produced in *ImageJ* using the following command: **Image>Stacks>Reslice [/]**… and then selecting ‘start at: top’ or ‘start at: left’ for the ‘16bitresliceYZ’ and ‘16bitresliceXZ’ data sets, respectively. The YZ reslicings also required mirroring in *ImageJ*: **Image>Transform>Flip Horizontally**.

The ‘8bitresliceXZ’ and ‘8bitresliceYZ’ represent the same data as ‘16bitresliceXZ’ and ‘16bitresliceYZ,’ respectively, that have been reduced from 16-bit depth to 8-bit depth using *ImageJ*.

#### Movies

For ease of navigation through entire image stacks, we also built self-contained compact movies in MPEG-4 format based on the 8-bit stacks mentioned above (Table 3). Most of the movies are slice stacks, but we also include movies of 3D volumetric models rotating about their orthogonal axes (Conf_RollSpinBodyMatrix.MP4; Arch_SlabSpin.MP4; Jeh_RollSpinBodySlab.MP4; Data Citation 1). Special image processing was performed to show the distribution of air spaces in the *Confuciusornis* amalgamation (CONVOID.MP4, Data Citation 1).

### Specimen Assays

#### Interpreting CT data

The grayscales in CT data reflect differences in X-ray attenuation that arise primarily from heterogeneous mass density and mean atomic number within the specimen, in addition to the X-ray spectrum employed in the scanning^47,60^. Individual CT slices are viewed as 2D pixel images, although each represents an actual volume that has been averaged down from the slice thickness. Slices are represented as grayscale images. Air is scaled as dark, but ideally not black, pixels. Our standard protocol at UTCT is to calibrate such that air has a positive data value, so no voxels have a value of zero. While making air ‘black’ may be cosmetically attractive, it results in a loss of information about the true location of material boundaries and the presence of scanning artifacts, in turn compromising the ability to make accurate measurements. White represents the highest density in the image, but in our data it is scaled on a sample-by-sample basis to correspond to the densest material present. As with air, we generally scale the data to avoid saturating any voxels (i.e., making them pure ‘white’), as doing otherwise results in information loss. In contrast to the variable scaling used for industrial-type CT data, medical CT data are usually calibrated to Hounsfield Units, which are largely reproducible between instruments. However, the Hounsfield scale was designed for medical imaging, and is inadequate for the dynamic range of materials found in geological specimens.

In all three datasets described here, the bone is denser than the matrix; that is, the bone appears in light-gray to (nearly) white, while the matrix is darker gray, and air is darkest. As noted, false color was added to certain images in *Photoshop* (Adobe Systems Incorporated) to highlight features described in the figure captions.

Using CT to assay an amalgamation is basically a 3D mapping exercise that evaluates the ‘fit’ between each pair of adjacent pieces separated by a fracture or grout joint. It involves identifying all individual pieces by mapping the fractures that separate them, and then conducting pairwise comparisons of 3D edge geometries of bones and rock fragments to determine whether adjacent pieces have a verifiable ‘fit.’ It also maps the distribution of consolidants such as grout and glue, which typically have their own unique grayscales (below). An additional concern is to identify all extraneous bone and rock fragments, that is, to identify any elements that do not have a verifiable association with the rest. The introduction of extraneous non-bone-bearing elements is a common practice to add structural integrity or to enhance appearances for display or for commercial sale. We refer to these as ‘shims.’ If left unidentified, extraneous elements can confound analyses of taphonomy and depositional environments related to the skeleton itself.

Fracture planes can take on myriad different orientations; hence pairwise comparisons are most effective when CT slice stacks are examined along all three orthogonal axes to map the boundaries of the individual parts in 3D. Objects are usually scanned along one axis, and the slice stacks for the other two axes are generated digitally. Powerful programs such as *VGStudio Max* (Volume Graphics GmbH), *Avizo*, and *ImageJ* enable this type of simultaneous examination (see Usage Notes). For convenience, we resliced the original datasets along XZ and YZ axes, and these datasets are provided in 16-bit and 8-bit versions (Table 2). We also assembled the resliced image stacks into self-contained MPEG-4 movies (Table 3).

#### Assay Rationale

In the three studies described here, the most informative evidence that CT data offered involved fracture geometry, and we mapped fracture surface trace geometry in map view, fracture face geometry in cross section, thickness and density of adjacent pieces, cross-cutting relationships among different generations of fractures, the continuity across fractures of bones and natural molds of bones that had fallen away from the slab, continuity of invertebrate burrows, and the distribution of grout and other consolidants. For other specimens, additional criteria such as bedding thickness and marker beds can be expected. The features we employed in this study have by no means exhausted those that CT can potentially reveal for validating other kinds of specimens.

#### Assay Procedures

The primary data produced in CT scanning the three specimens described here were sinograms^47,60^. The sinograms were then convolved in a process referred to as ‘image reconstruction’ in which the sinograms are converted into a sequence of cross-sectional images of the scanned object. The cross-sections display on a computer screen as pixel images, but because each pixel actually represents the slice thickness averaged down into a 2D plane, these images actually represent voxels. Thus, the primary data used for interpretation of the scanned object are voxel images, and the datasets represent large cylinders (‘bricks’) of voxels.

Once each specimen had been scanned, we created a 3D ‘volumetric rendering’ or ‘volume model’ from the original CT dataset using the volume rendering software *VGStudio Max* or *Avizo* (see Usage Notes). In an ongoing discussion of optimal imaging methods for fossils, a large contingent of paleontologists prefers the visual effects produced by ‘surface renderings’ or ‘surface models’ of a scanned object. Surface rendering produces a fine mesh of polygons that describe the object’s external and internal surfaces, or the external surface of some region of interest. But the volumes between those surfaces are simply empty space when visualized as surface renderings. There are many advantages of surface models. They afford a standard format for 3D printing, and they produce smaller files than the original full-resolution voxel dataset that are easier to manipulate on inexpensive computers.

For forensic evaluation, we emphasize the superiority of volume rendering over surface modeling. Surface modeling can indeed generate visualizations that resemble those produced by volume rendering, but they effectively discard the original voxel data, and are therefore less amenable to secondary verification. In forensic analysis, as well as for many conventional scientific goals, data validation is a requisite step in the research protocol, hence our preference for volume rendering, and our decision to make the original voxel data available (Data Citation 1). Interpreting the structural integrity of an object is fundamentally a 3D problem, and internal volumes are rich terranes for scientific discovery, at least in authentic specimens. Volume renderings work by mapping each voxel value to a color and an opacity value, allowing some voxel values to be rendered transparent or semi-transparent and others to be rendered opaque. Surface models can produce similar visualizations, but volume renderings preserve the density information contained in the original CT data. The greater cost of volume rendering is that voxel datasets are generally large and computationally demanding to work with. Typically they are visualized as images of external surfaces, cross sections, or 3D cutaway views and only a small proportion of the total dataset is visualized in any single image. Their overwhelming advantage for the forensic analysis described here is that subsequent users can validate for themselves adjustments that were made to the threshold and range of displayed densities used to reveal fractures and cosmetic applications, and otherwise invisible internal structure.

This point is illustrated by comparing a surface model (Fig. 2a) and a volume rendering (using the Phong algorithm) of the *Confuciusornis* dataset (Fig. 2b). The surface rendering is no better than conventional photography (Fig. 2; Supplementary Figures) in revealing the fracture pattern of the top slab, whereas volume rendering reveals vividly that the surface layer was shattered. By adjusting the display threshold, the surficial grout and paint could be filtered away in the volume model to see the underlying structure.

In our volume renderings, in map-view one can see where edges of separate pieces are either tightly or poorly aligned on either side of a fracture. In the *Confuciusornis* amalgamation, false ‘repair’ of the mandible is evident where a square corner of an extraneous piece is set against a curved face of the adjacent piece (Figs 5 and 6). One can also see in this image twirled ‘strings’ of grout that were set in to hold the extraneous piece at its edges. The absence of matching edges between adjacent pieces is even more obvious in the Archaeoraptor amalgamation, in the center of the slab where the tail was joined to the body in a jumble of mis-matched pieces (Fig. 9).

A map view of the volume rendering also shows where marked differences in density or thickness exist between adjacent pieces, suggesting a misfit and/or admixture of an extraneous piece. This can be seen in the Archaeoraptor amalgamation (Fig. 9) between pieces labeled 5a/5b, which are relatively dark (i.e., less dense) and the adjacent pieces labeled L and M, which are brighter (i.e., denser).

In cases where the bone and matrix are of non-overlapping densities, both surface and volume rendering offer the ability to digitally filter out surrounding matrix to visualize the entire skeleton. The *Confuciusornis* amalgamation shows this well, where volumetric filtration of the dataset was used to isolate the semi-articulated skeleton from its encasing matrix (Conf_PitchSpinBodySkel.MP4; Conf_RollSpinBodySkel.MP4; Data Citation 1). This enabled examination of the skeleton from all angles to evaluate the positioning of the bones with respect to one another, and provided informative evidence for judging the authenticity of the skeleton as a whole. This process was less successful with *Jeholodens* and Archaeoraptor because bone and matrix overlapped in density.

The volume rendering of *Confuciusornis* also added considerable scientific value to the specimen by revealing all of the surfaces that remain concealed by matrix as well as providing a foundation for quantitative analysis of individual bones. Additional bones unexposed on the slab face are present in the underlying mudstone, for example cervical vertebrae that lie beneath the skull (Conf_RollSpionHeadSkel.MP4, Conf_YawSpinHeadSkel.MP4, Data Citation 1). In certain cases, this type of digital preparation, using either volumetric or surface rendering, can provide a satisfactory and cost-effective alternative to conventional mechanical preparation. Lastly, one can potentially convert the matrix-free volume rendered skeleton into a surface model for 3D printing.

Volume rendering was used as a base map for identifying the fractures and separate elements of the *Confuciusornis* (Fig. 5) and Archaeoraptor amalgamations (Fig. 11). It was exported as an image of each slab surface into *Illustrator* (Adobe Systems Incorporated), where the surface bones and fractures were traced onto different ‘layers’ of the *Illustrator* file. The map was generated by tracing the fracture pattern in surface view, while simultaneously evaluating the geometries of adjacent pieces in cross sections.

Cross sections of the *Confuciusornis* amalgamation (Conf_XY.MP4, Conf_XZ.MP4, Conf_YZ.MP4; Data Citation 1) show it to be less crushed than it appears on the surface. Some of the seemingly flattened bones are merely pressed into the underlying sediment, and some still preserve natural hollow cavities. In cross section one can also see that the larger limb bones, although hollow in life, were crushed during burial, shattered during excavation, and several different elements flaked away and were glued back together during assembly of the amalgamation. A thin dark line of separation beneath bone fragments and the rest of the slab is indicative of the low-density consolidant used in these repairs (Fig. 4, arrow).

The cross sections also proved most decisive in evaluating whether pairs of assembled pieces were lying in natural relationships to one another. The most important criteria in validating the adjacency of pieces include matching thickness, density, fracture face geometry, and the distribution of grout. This is illustrated for the *Confuciusornis* amalgamation in Figs 3,4,5,6, and for the Archaeoraptor amalgamation in Fig. 8. In the latter, Slice 30 (Figs 9a and 10) crosses a portion of the slab in which all but one of the pieces lie in natural relationships to each other. Pieces 1a, 1b, 1d, and 1e are not separated by grout and are matched across fractures in thickness, density (brightness), and fracture face geometry. Piece FF lies adjacent to Piece 1e, however the latter is thicker and denser than the former. The grout has fewer bubbles than elsewhere, and its distribution beneath Piece FF suggests that this is an extraneous shim that was grouted onto the slab toward the end of assembly of the amalgamation (see Arch_XY.MP4; Arch_XZ.MP4, and Arch_YZ.MP4; Data Citation 1).

Slice 220 (Fig. 9b) of the Archaeoraptor amalgamation crosses a portion of the slab that was entirely fabricated from unverifiable pairs of pieces, as evidenced by the distribution of grout between pieces of mismatched thickness and density, the lack of similarity in fracture faces, and the lack of continuity in the bones. This is the region of greatest unconformity, involving admixture of the extraneous tail fragments and the shards of rock that still adhere to these small, broken bones. In cross section, all pieces containing caudal vertebrae are far thinner than the surrounding pieces, there are no matching geometries between fractured edges, and grout separates most edges from the rest of the pieces comprising the surface layer (Figs 9 and 10).

Geological events that preceded discovery can leave natural evidence that is informative in pairwise comparisons between pieces assembled into an amalgamation. This is most evident in the Archaeoraptor amalgamation where at least three separate fracture episodes are apparent. One fracture event can be identified as the oldest, and was probably caused by an ancient earthquake or tectonic unloading as the buried lakebeds returned to the surface. It is marked by the deposition of a dense material, probably carbonate, that was absorbed into adjacent fractured edges as groundwater circulated through the fractures. The result is that these edges have ‘stripes’ of higher density and greater brightness than the edges of rocks fractured at some later time (Fig. 9: slice 30, Pieces 1b-1d).

The second fracture event was either natural or occurred during excavation as the specimen was shattered. In Slice 30, pieces 1d and 1e can be matched across fractures in thickness, density, fracture face geometry, and they are not separated by grout. They also lack the carbonate ‘stripe’ associated with the first-generation fractures. A mismatch is evident when a piece bearing the bright carbonate stripe is aligned adjacent to a piece lacking the stripe. This can be seen in the map view volume rendering of Archaeoraptor (Fig. 8), and confirmed in Slice 220 (Fig. 9), where Piece F lacks the carbonate stripe but it is positioned adjacent to piece 5a that has the carbonate stripe. There is also grout between the two, strengthening the diagnosis that Piece F is extraneous and was added late in the construction process.

A third set of fractures can be seen in both surface and cross sectional views, in which fractures propagated but did not rupture into separate pieces (Fig. 9). This almost certainly happened during discovery, and/or later as the grout set. In the *Confuciusornis* amalgamation some of the top layer fractures are continuous with fracture patterns in the grout, and may have been caused by differential shrinkage of the grout as it hardened (Figs 4,9).

Additional fractures are manifest in delamination of the shale beds comprising the backing slab, and these are more certainly attributable to differential shrinkage of the grout as it hardened. In the *Confuciusornis* amalgamation delamination separations are evident in both the backing slab and surface slab. These voids are artifacts of the construction technique, and can be seen in 3D in a Supplementary movie (CONVOID.MP4, Data Citation 1).

One short invertebrate burrow was evident in the Archaeoraptor amalgamation. It runs for a few millimeters horizontally through the sediment beneath the skull, and crosses from Pieces 1b-1d and matches across the fracture separating these two pieces. It is visible in XY slices 10–16 (Arch_XY.MP4; Data Citation 1). These have proved highly informative in understanding the geological history of other fossils that we have scanned.

The ceramic grout used to consolidate both the *Confuciusornis* and Archaeoraptor amalgamations has a very distinctive texture. Its signature is lower density than the top and backing slabs, containment of air bubbles and tiny metallic inclusions, and a unique fracture pattern. The grout also has an inconsistent pattern of adhesion to the slabs on either side, often becoming detached over large regions and leaving voids. Air bubbles in the grout show up as dark voids. Bubbles are introduced when powder and liquid are mixed, and the distribution of bubbles is informative of the firmness of the grout when applied. In relatively less saturated and fresh mixtures, the bubbles tend to rise and become trapped against the bottom surface of the top slab pieces. As the grout stiffens, the bubbles become less mobile. The history of application can be mapped with the aid of bubble distribution (Fig. 11). Different mixtures of grout can be identified by the different sizes and clustering patterns in bubbles.

Metallic inclusions within the grout are the brightest and densest objects in the amalgamation. Tiny metallic fragments are a common contaminant in man-made objects such as these, generated as metallic tools are used and sharpened.

We note in passing that delamination is an indication that this method of repair introduces its own destabilizing influence to whatever authentic pieces are present, and that other consolidants should be sought if the production of amalgamations remains the preferred method for reassembly of fossils from thinly bedded sediments that are broken or shattered during excavation.

Finally, we note that there are two artifacts that affected the dataset, one being the ring artifact that results from inhomogeneity in individual detector crystals during scanning. The second artifact is called the ‘longhorn artifact’ and it is a result of the geometry of the object, which is wide and flat. This means that the X-ray beam has a very short course when the specimen was perpendicular to the beam, but a very long course when parallel to the beam.

#### The role of photography

Photography is a staple in all manner of forensic documentation. Many paleontological publications submit photographic documentation in defense of the authenticity of the described specimen. However, in revealing the nature of the *Confuciusornis* amalgamation the superiority of volume-rendered CT data over any of the various reflected light imaging techniques is clear by comparing Figs 2,3,4 (see also Supplementary Figures). Figure 2 is a color photograph. Supplementary Figs 1–6 are conventional black-and-white photographs using fine-grained professional film of the top surface. Neither was effective in illuminating the full degree of shatter-fracturing of the bone-bearing top layer, or in mapping the many parts of the amalgamation (Figs 5,6) owing to the cosmetic treatment that the surface had received.

Both color and ultraviolet light photography of the Archaeoraptor amalgamation (http://digimorph.org/specimens/Archaeoraptor_forgery/) were more effective in showing that it too was a shatter-fractured amalgamation built of heterogeneous elements. UV photography performed best in brightly illuminating the bone, and in showing different reflectance of pieces with different densities. For example, Pieces 5a-b reflected UV light more brightly than the adjacent mis-matched Pieces J and K (see Fig. 10). The grout tends to fluoresce a green color under UV light thus highlighting the fracture pattern where separate pieces were grouted together, but conventional photography offered a cleaner picture of all of the fractures. The heterogeneous reflectance of surface pieces in UV photography is symptomatic of an amalgamation and an indication that CT scanning will probably reveal much more of the object’s forensic history. Additional photographs of all three specimens described here are available in the Supplementary Figures, for anyone wishing to make more detailed comparisons of these techniques.

UV light was recently used to propose that soft tissues were preserved in the type specimen of *Micoraptor gui*^61^. However, the distribution of fluorescence in this specimen corresponded closely to grout in photographs of the specimen^17^, in what appears to be another amalgamation. CT scanning this specimen can potentially resolve doubts about whether the fluorescence is an artifact of amalgamation reassembly, rather than evidence of soft tissue preservation. More recently, UV laser stimulated fluorescence revealed the skull of a different *Microraptor* specimen to be a composite of at least two different specimens^62^. However, these authors note the possibility that differential fluorescence may be the result of taphonomic effects. Here too, CT scanning can potentially resolve any doubt concerning the composite nature of this specimen.

We note that CT failed to document any integumentary structures (feathers, hair). If present, their remains are too thin and too low in relative density to register in the scans. In such cases, photographs are superior in documenting surficial features of the slabs. However, CT can decisively resolve whether extraneous pieces have been joined together and where grout has been applied. Thus, both photography and CT have their places in forensic documentation and analysis.

### Assay determinations

#### *Confuciusornis*

As can be seen in the photographs (Fig. 2, Supplementary Figures), this object appeared to be a complete articulated skeleton, preserved intact on the surficial bedding plane of a solid slab of shale. Analysis revealed that the specimen was shattered during excavation, and reassembled on an uneven bed of grout applied to the surface of a solid slab of shale. Grout and paint were also applied over much of the surface around the skeleton to obscure the shatter-fracture pattern. Grout was also applied along the edges of the slab, obscuring the three-layered stratigraphy indicative of a man-made amalgamation. The top layer including the bone is quite thin, ranging between 2 mm to a maximum of~8 mm. The grout layer ranged from ~3 mm to ~6 mm, and the entire amalgamation was between 20 mm to 25 mm thick.

The CT scans revealed that the surface layer of the amalgamation was assembled from 81 separate pieces. They can be classified into three groups with more or less separate histories. There are 22 associated bone-bearing pieces which contain most of the skeleton, and they were correctly reassembled to reveal a skeleton in its natural death posture (Confu_PitchSpinBodySkel.MP4, Confu_RollSpionBodySkel.MP4; Data Citation 1). The most glaring absence is any evidence of a sternum, which was probably lost during excavation. An additional 55 extraneous shims with no bone or verifiable relationship to the skeleton were introduced during construction.

The last four elements of the amalgamation surface consist of bone-bearing pieces that lacked verifiable association with the skeleton and were classified as extraneous. Two had been placed at the back of the mandible (Figs 5,6), where the skull was badly fractured during excavation. The anatomical effect presents an unusually large mandibular fenestra of unique construction bordered inferiorly by a thin splint of bone. In contrast, other specimens of *Confuciusornis* have a small fenestra bordered by a broad plate of bone^56^. Mismatching outlines are evident in surface view once the obstructing paint was filtered away (Figs 3,6); in cross section the joint faces are dissimilar and the pieces are of considerably different thickness, the extraneous pieces being thin flakes (Fig. 6b,c). ‘Strings’ of grout lying between these and the adjacent pieces indicate that they were set into place late in the assembly process.

A third extraneous piece was added to the left hand, and seems to represent the distal phalanges of left digit III. Here too, the absence of matching fracture planes and differing thickness in cross section suggests that this piece is out of place and may not have come from this specimen at all. The fourth non-verifiable piece may be a fragment of right manual digit II that broke away during excavation and was later glued to the surface.

The skeleton is preserved lying on its back. The skull is bent forward and down over the long neck in such a way that its dorsal and left sides lie exposed on the slab face. The beak covers all but the anterior-most cervical vertebrae; in the CT scans the remaining cervical vertebrae can be seen preserved beneath the skull. Partial dorsal surfaces of palatal bones are visible through the right orbit. The right mandible is exposed in lateral view, disarticulated from the cranium but still lying close by. It was reconstructed with two extraneous pieces. The palate, basicranium, and left mandible are buried beneath the other skull bones and are not visible on the slab’s surface.

The axial skeleton was slightly disarticulated during decomposition and shortly after burial. Several of the mid-cervical vertebrae have drifted away from their natural positions. The base of the neck is separated and dislocated well to the right of the dorsal vertebrae. The base of the tail is separated from the sacrum, and the proximal caudal vertebrae have drifted away from their natural positions and come to lie in a disorganized pile near the distal end of the pubes. Much of the cervical and dorsal vertebral column lies obscured beneath the overlying bones of the skull and arms, but they can be seen in the CT scans by filtering the matrix away. Several mid-dorsal vertebrae are partially exposed in lateral view. The remaining dorsal centra are exposed in ventral view, as are the sacrum, distal caudal vertebrae, and pygostyle. Most of the ribs have also drifted slightly from their natural positions and lie flat on the bedding plane. Short segments of very tiny ribs, possibly representing gastralia, lie in unnatural positions between the pubes.

Prominently visible on the face of the slab is a large furcula. Parts of the right and left coracoid, and the proximal end of the right scapula can also be seen, but their exact shapes are obscured by matrix. The left forelimb is extended slightly outward from the body, with its ventral or palmar surface facing up. Elements of what might be digit III are present, but their association with the skeleton cannot be verified (below). The right forelimb is folded at the elbow joint to lie over the thorax such that the forearm and hand cover much of the humerus. Several phalanges in each hand are slightly displaced. There is no evidence of a sternum.

The cranial surfaces of the pubes are visible from their proximal ends to their fused distal symphysis. The cranial edges of both ischia are exposed between the pubes, but little of their overall form can be seen. Both ilia are entirely encased in matrix. Both femora are shattered and lie dislocated from the hip sockets. The tibiae are also crushed. The fibulae form thin splints of bone extending from the knee joint to a point midway down the shaft of each tibia. The proximal tarsal bones appear fused to the tibiae, but matrix obscures details of the relationships among these bones. The metatarsals appear fused to the distal tarsals, and the proximal ends of the metatarsals themselves appear to be fused. The left foot is intact and has a reversed hallux that lies close to the distal end of the second metatarsal. The right foot is complete but its digits are slightly displaced, and its first metatarsal is hidden from view.

#### ‘Archaeoraptor’

As presented for scanning, it appeared to be a complete, articulated skeleton preserved on the surficial bedding plane of a more massive slab of shale. No counter slab was associated with it. Its surface layer was built from 88 individual pieces and reassembled as a mosaicked amalgamation. Little of its surface had been painted or grouted, and the fracture pattern was more clearly visible than in *Confuciusornis*. Nevertheless, conventional preparation of its surface carried out at the Tyrell Museum failed to reveal its composite nature.

The top layer including the bone is somewhat thicker than the *Confuciusornis* amalgamation, ranging between ~4 mm—to a maximum of ~10 mm. The grout layer ranged from ~2 mm to ~5 mm, and the entire amalgamation was 27 mm at its thickest. The top layer pieces can be classified into three groups with more or less separate histories of construction. The first group to be assembled comprises roughly one-third of the slab surface and includes 23 pieces that lie, as reconstructed, in natural association with one another (Fig. 10). Together, these pieces contain roughly the anterior half of a semi-articulated skeleton of a new species of ornithurine bird, later referred to the taxon *Yanornis martini*^16^. The skull lies flattened on its left side, and the exposed right side shows a row of teeth in the maxilla and dentary. The cervical vertebrae are slightly disarticulated, but the head and neck are positioned in hyperextension characteristic of rigor mortis. The presacral vertebral column is partly exposed along with several right ribs. The right wing is flexed with the hand lying over the thorax. The left wing is partially extended. A large furcula and a robust sternum with a tall keel are present. Little of the pelvis is preserved. The shaft of the right femur is partially preserved.

The second group of surface layer pieces includes 26 pieces containing ‘associated bones’, plus a few barren pieces that are verifiably contiguous (Fig. 10). These bones are arranged to portray the articulated rear half of the skeleton, but they were added secondarily and none preserves evidence of a natural attachment to any piece of the first group. The third category includes 39 shim pieces that lack bone entirely, or contain only dissociated small fragments of bone (Fig. 7d). The shims vary in thickness, density, and color, and generally are separated from the other pieces by seams of grout. None preserves evidence of a natural attachment to any piece of the first or second group. Shims were added to secure the ‘associated bones’ into position around the bird skeleton and to make the slab more presentable.

The sequence of assembly (Fig. 11) was probably as follows. The partial *Yanornis* skeleton was assembled first and the ‘associated bones’ were positioned secondarily. The first associated bone to be added to the bird skeleton was a longitudinally split femur, positioned as if it is the left that accompanies the right in the bird skeleton. Similar profiles and surface staining suggest this might be the counter piece to the right femur of the bird skeleton, but severe damage to both prevents verification. The next pieces to be added were badly fragmented apparent right and left tibiae and fibulae. They are probably both from the left side and represent the part and counter-part of a single articulated tibia-fibula. Next were the apparent right and left feet, again, part and counter-part of a single right foot. The articulated distal end of a tibia is preserved in the same piece with the foot, but is rotated 90^o^ from the orientation of the more proximally situated tibia-fibula. This indicates that pieces containing the foot probably came from a different specimen than the tibia-fibula.

The last bones to be added were several fragments lying behind the bird pelvis, followed by the elongate stiff tail. The tail is broken into five discontinuous pieces set against thin flakes of matrix that float on thick grout seams. It consists of approximately 20 distal caudal vertebrae that are preserved with elongate rod-like extensions of the prezygapophyses and chevrons characteristic of non-volant dromaeosaurids. This tail was reported as part of the type specimen of *Microraptor zhaoianus*^11^, however, the basis for this assertion was never explained, and this association is no better validated than its prior association with the partial *Yanornis* skeleton.

The associated bones do not duplicate any bones found in the bird skeleton, but they introduce several phylogenetic character conflicts. The tail of a non-volant dromaeosaur is incongruous with the ornithurine characteristics of the shoulder girdle and wing, and also with the foot, which lacks the characteristic enlarged second claw of dromaeosaurids. Apart from size and hollowness, there is no evidence to suggest that the femur, tibia/fibula, foot or tail belong to either the same individual or species. Taken collectively, the Archaeoraptor slab evidently represents two or more species, assembled from at least two, and possibly five, separate specimens^6^.

#### *Jeholodens*

The *Jeholodens* dataset (Jeh_RollSpinBodySlab.MP4; Jeh_XY.MP4; Jeh_XZ.MP4; Jeh_YZ.MP4; Data Citation 1) was included to give an example of a nearly perfect, unfractured specimen that shows minimal evidence of human intervention (Figs 12,13). It is a much smaller specimen than the *Confuciusornis* and Archaeoraptor amalgamations, and this contributed to its intact preservation upon discovery. The dataset shows far fewer features than the others, largely because they are amalgamations and their history of human construction is reflected in a complex man-made stratigraphy, the reassembly of numerous broken pieces, and the introduction of extraneous pieces into a mosaicked composite.

The *Jeholodens* specimen was discovered intact and preserved between the split part and counter-part, with most of the skeleton remaining on the part that we scanned. Within this small slab, delamination between bedding planes beneath the skull was consolidated with an unidentified type of glue that penetrated deeply into the fracture (Fig. 14). Owing mostly to its small size, this specimen was not shattered in the process of discovery and did not need the additional structural reinforcement of an extraneous backing slab. We present it here as a rare example of an intact specimen, damaged only slightly as it was split into part and counter-part, and showing only very subtle evidence of repair.

At its maximum, this tiny slab is 10.2 mm thick. Bright streaks perpendicular to the bedding planes can be seen in cross section (Fig. 14). Most are confined to the lower third of the slab, but a few cross its entire thickness. These represent natural fractures that were later diagenetically healed by a denser material, probably a carbonate.

This specimen consists of a partial skull and complete postcranium. Most of the skeleton lies flattened on the surface of a bedding plane along which the part and counterpart separated. The skeleton is lying on its ventral surface with the right forelimb folded beneath the rib cage and the hand partially disarticulated. The other limbs are outstretched, and the elements of the hand and feet are slightly separated from natural articulation. The tail is curled. The skull and postcranium were flattened and crushed during burial. It is peculiar in that the fibulae lie medial to the tibiae, a presumed taphonomic artifact.

We noted above that this CT data set become noisier toward the back of the specimen. The slab is triangular, and the increased noise is a reflection of the longer path the X-rays tooktoward the rear of the block.

## Data Records

### Original *Confuciusornis* Data Folders

University of Texas High-Resolution X-ray CT Facility Archive 0044, 0109 & 0110.

### Original ‘Archaeoraptor’ Data Folders

University of Texas High-Resolution X-ray CT Facility Archive 0122.

### Original *Jeholodens jenkinsi* Data Folders

University of Texas High-Resolution X-ray CT Facility Archives 0100, 0101, 0555.

See Tables 2 and 3 for a description of samples and data output, and for a list of movies available for download.

## Technical Validation

The primary issue requiring validation involves the spatial geometry of the CT imagery as reconstructed by the ACTIS software that operated the scanner. Each scan is preceded by a calibration to identify precisely the center of rotation of the turntable with respect to the chosen detector. We also periodically undertook data calibrations by scanning complex objects (‘phantoms’) whose dimensions were known with precision, and compared spatial data in X-, Y-, and Z-axes in the imagery generated.

Calibrations are also necessary to establish the characteristics of the X-ray signal as read by the detectors under scanning conditions, and to reduce geometrical uncertainties. The latter calibrations vary widely among scanners; as a rule flexible-geometry scanners such as the one at UTCT require them, whereas fixed-geometry scanners geared towards scanning a single object type may not.

The two principal signal calibrations are offset and gain, which determine the detector readings with X-rays off, and with X-rays on at scanning conditions, respectively. We used an additional signal calibration known as a ‘wedge’ that consists of acquiring X-rays as they pass through a calibration material over a 360° rotation. The offset-corrected average detector reading is then used as the baseline from which all data are subtracted. If the calibration material is air, the wedge is equivalent to a gain calibration. A typical non-air wedge is a cylinder of material with attenuation properties similar to those of the scan object. Such a wedge can provide automatic corrections for both beam hardening and ring artifacts, and can allow utilization of high X-ray intensities that would saturate the detectors during a typical gain calibration^60^.

## Usage Notes

Many image processing programs can be used to manipulate these datasets. The following are those that we used, and we include instructions for performing common tasks. The various conversions of the original datasets (Table 1; Data Citation 1) were carried out using these procedures.

### Programs for Image Processing

#### Freeware

1. ***IrfanView*** (http://www.irfanview.com/). Useful for viewing serial slice sequences, renaming and resizing files, and converting file formats
2. ***ImageJ*** (https://imagej.nih.gov/ij/). Can be used for viewing image stacks individually or along all three orthogonal axes simultaneously. Used for image processing (e.g., cropping, adjusting levels, reslicing data, adding slice numbers) and measuring. *ImageJ* offers many other useful features, and the above list is far from comprehensive—we encourage self-guided exploration of this powerful software. Because *ImageJ* is open-source, there are also many useful plugins that can be downloaded from third-party webpages. For example *BoneJ* is a plug-in that provides tools for trabecular analysis and whole bone shape. Some plug-ins are linked from the *ImageJ* website, and a version of *ImageJ* called *Fiji* is available that includes most of the available *ImageJ* plugins. Among the plugins included with an *ImageJ* standard download are several that can be used for volume rendering or generating surfaces with tomographic data. Although these 3D Plugins are slow, and may sometimes fail on large data sets, they may provide a useful free alternative for generating static images if *Avizo* or other 3D packages are not available.
3. ***3DSlicer*** (https://www.slicer.org/). 3D visualization and measurement program (for those who do not want to buy a 3D visualization program).
4. ***Drishti*** (http://sf.anu.edu.au/Vizlab/drishti/). Volume exploration and presentation tool.
5. ***SPIERS*** (http://spiers-software.org/). A useful software toolkit for tomographic visualization that generates surface models.
6. ***Blob3D, Quant3D, Align3D, and MuCalc Tool*** (http://www.ctlab.geo.utexas.edu/software/). UTCT has developed a number of specialized software packages for quantitative analysis of HRXCT datasets. *Blob3D* measures 3-D geometric information on up to thousands of discrete objects within a data volume. *Quant3D* quantifies three-dimensional fabrics using a variety of metrics. *Align3D* performs a three-dimensional alignment and subtraction of two data sets, allowing differences between them to be determined and analyzed precisely. *MuCalcTool* is a Microsoft Excel workbook for computing and comparing the X-ray attenuation of various minerals at different X-ray energies. All of these programs are freely available for academic use.

### Commercial 3-D Visualization & Measurement Software

1. ***VGStudio Max*** (http://www.volumegraphics.com/en/). A relatively expensive but powerful program, excellent for making volumetric renderings, segmenting and making surface models.
2. ***Avizo*** (formerly Amira) (http://www.fei.com/software/avizo3d/). A less expensive product than *VGStudio Max*, excellent for measuring, segmenting and making surface models. *Avizo* currently has the best suite of segmentation tools available.
3. ***Mimics*** (http://biomedical.materialise.com/mimics). Software specifically developed for image processing of 3-D data from CT, MRI, micro-CT, CBCT, 3-D Ultrasound, and Confocal Microscopy.

### Animation Software

1. ***QuickTime Pro*** (www.apple.com/quicktime/download/). Excellent software for producing self-packaged animations from slice stacks or image frames.
2. ***Windows Live Movie Maker*** (http://windows.microsoft.com/en-us/windows/movie-maker). An alternative to *QuickTime* that tends to make movies with larger file sizes for a given resolution and quality level, but in formats that may compatible with other programs (e.g., PowerPoint).
3. ***Blender*** (https://www.blender.org/). A free and open-source 3-D creation suite. It supports the entirety of the 3-D pipeline—modeling, rigging, animation, simulation, rendering, compositing and motion tracking, even video editing and game creation. This is a sophisticated presentation tool, but is not aimed at scientific data analysis.

### PROCESSING WITH *ImageJ*

#### Resizing and Cropping Dataset

For convenience, we provide cropped and resized versions of all three original datasets (Table 1, Data Citation 1). We also offer the following instructions for several common procedures that are useful in working with other datasets.

Reducing the size of a dataset can greatly speed its manipulation in 3D rendering programs. This is especially true for memory-intensive activities like segmentation. Simple ways to reduce the size of a dataset are to down-sample the data from 16-bit to 8-bit TIFFs (reduces file size by 50%), downsize the dimensions of the slices (e.g., from 1024×1024 pixels to 512×512 pixels; reduces file size by 75%), and/or crop the slices to remove empty canvas.

Another reason to convert from 16-bit to 8-bit is that many programs cannot handle 16-bit data, or offer only limited options for processing them (e.g., *Illustrator*, *Photoshop*, *IrfanView*). For example, *Photoshop* reads in 16-bit data as 15-bit data (although all documentation refers to it as 16-bit), resulting in a small amount of data loss that may affect some users (e.g., those using the data in numerical analysis programs or simulations). Therefore, we strongly recommend doing 16-to-8-bit conversions using *ImageJ*, *Avizo*, or *VGStudio Max*.

**To load a 16-bit image sequence into *ImageJ* use these menus. File>Import>Image Sequence.** Then navigate to the 16-bit folder and double click on 1st slice; select **OK** in **Sequence Options** window. Or, drag and drop folder containing 16-bit images to ImageJ control panel.

If *ImageJ* prompts that you do not have enough memory to open the stack, then you will need to change the amount of memory allocated to ImageJ using the following menus: **Edit>Options >Memory & Threads.** Then increase **Maximum memory** to a larger value (corresponding to your computer’s capacity). Close and reopen program. For computers lacking sufficient memory, image sequences can be loaded as virtual stacks.

**To down-sample the data, go to: Image>Type>8**-**bit**. *ImageJ* will convert the open images to 8-bit. Note that this operation optimizes the grayscales to whatever range is displayed in the viewer. If you wish to maximize the range of grayscale values when converting from 16-bit to 8-bit, then the entire volume needs to be set to the maximum 16-bit range prior to down-sampling to 8-bit: **Image>Adjust>Brightness/Contrast.** In the window that pops up, click **Set**, and adjust the **Minimum** and **Maximum displayed value** to **0** and **65535** respectively (or, for ‘Unsigned 16-bit range’ select ‘16-bit’).

#### Resizing and Cropping Images

*ImageJ* allows images to be resized and cropped. *We caution that* cropping images (below) does not change in-plane resolution, but resizing does. To resize the data, select: **Image>Adjust>Size.** Set new width and height as desired (e.g., 512 pixels), and select OK. It is advantageous to pick a size that is a multiple scalar of the original dimensions as this will require no interpolation of the data.

To crop the data, select a crop area by clicking-and-dragging the mouse on an image in the stack. Check each side of cropping bounding box with the slider through the entire image sequence to make sure specimen is not clipped. Select: **Image>Crop**. After down-sampling, resizing or cropping the data, select: **File>Save As>Image Sequence.** Create a new folder into which the image sequence is saved.

#### Orthogonally Reslicing Data

For convenience, we provide orthogonally resliced datasets (Data Citation 1) that were processed using ImageJ using the following steps: **Image>Stacks>Reslice [/]**: in the pop-up window, choose the side of the stack from which you wish to reslice (e.g., Top, Left, Bottom, Right) and click **OK**. *ImageJ* will indicate the direction of reslicing as it processes the data, and will generate a resliced stack. Then select **File>Save As>Image Sequence (**make a new folder and save the resliced data).

Note that ImageJ exports the reslicings with a voxel aspect ratio of 1:1:1, so the YZ reslicings will look ‘squatty’ and the XZ reslicings will look ‘stretched’ if the Z (interslice) spacing differs from the X and Y (interpixel) spacing (either because the original voxels were not equant, or because the data have been resized). In such cases, the reslicings require stretching to their appropriate aspect ratio. To do this, first calculate the voxel aspect ratio: interslice spacing/(field of reconstruction/image resolution). Then multiply voxel aspect ratio by the number of slices in the original data; this gives you the correct length, in pixels, for your reslicings; round to the nearest pixel. In *ImageJ*, open one of your reslicings if it is not already open, then select: **Image>Scale**: in the pop-up window, adjust the **Width (pixels)** or **Height (pixels)** to the appropriate length as calculated above. To close: **File> Save As>Image Sequence**.

#### Adding Slice Numbers

We recommend numbering each slice so that subtle features can be referenced and indexed. To do this in *ImageJ* use the following menus: **File>Import>Image Sequence:** browse to appropriate folder and load slices. Next: **Image>Stacks>Label:** starting value is 1, interval is 1, X location is 5 (pixels from left margin), Y location is 18 (pixels from top margin), font size is 14, and text is blank. Click **OK**. These are our standard values for DigiMorph.org processing. Feel free to change the location of the labels as desired. If you cannot see the slice numbers, the text is likely black (*ImageJ*’s default). Change the text color to white by going to: **Edit>Options>Colors**: select white as the foreground color. Then repeat the step above to add the label. To close: **File>Save As**>**Image Sequence**.

#### Orthogonal Views

This option presents a dynamic reslicing of the original CT slice stack. The ‘cross-hairs’ can be dragged with the mouse to locate features in the different planes. Note that, as with the resliced data, the orthogonal views assume equant voxels, and the reslicings will look stretched or squatty if the voxels are not isometric. Go to: **Image>Stacks>Orthogonal Views.**

#### Histogram

This function allows histogram measurement of the grayscale values in one slice, the whole volume, or some region of interest (as defined with the selection tools). This is an easy way to quantify the distribution of grayscale values in your data. Select: **Analyze>Histogram.**

#### Adjust Brightness and Contrast

To change the contrast or grayscale levels of your data, we recommend adjusting the brightness and contrast on the 16-bit data prior to downsizing to 8-bit—this will retain far more data than adjusting after converting to 8-bit data. Note that *ImageJ* uses the adjusted window levels of the 16-bit data as the grayscale range for the down-sized 8-bit data (see above discussion). Select: **Image>Adjust>Brightness/Contrast**

#### Measuring

Will measure lengths, areas, grayscale values (including standard deviation; mean, min and max values), angles, coordinates of points, and a variety of other values, depending on the chosen selection tool. Select: **Analyze>Measure.**

#### Line Profile Plots

Will plot the grayscale profile of a line or rectangle. Select: **Analyze>Plot>Profile.**

#### Binning Data

This will downsample the data in X, Y, and Z planes. Select: **Image>Transform>Bin.**

#### Transform Data

Allows rapid reorientation and downsizing of the data (i.e., flipping, rotating, translating, binning, etc.): **Image>Transform**: choose appropriate transformation.

## Additional Information

**How to cite**: Rowe, T. B. *et al.* X-ray computed tomography datasets for forensic analysis of vertebrate fossils. *Sci. Data* 3:160040 doi: 10.1038/sdata.2016.40 (2016).

## Supplementary Material

Supplementary figures

## Figures and Tables

**Table 1:** Scanning Parameters.

	***Jeholodens***	***Confuciusornis***	‘**Archaeoraptor**’
X-ray source setting*	250 kV, 3.5 mA	420 kV, 4.4 mA	420 kV, 4.7 mA
X-ray focal spot size	1.0 mm	1.8 mm	1.8 mm
Detector†‡	RLS	P250D	P250D
X-ray pre-filter§	None	2 brass plates (1.58 mm each)	1 brass plate (1.58 mm)
Wedge||	Air	Air	Air
Offset¶	0%	160%	190%
Source-object distance#	752 mm	710 mm	700 mm
Views**	1800	1200	2000
Rays averaged††	2	1	1
Samples per view‡‡	1	1	1
Integration time‡‡	25 ms	64 ms	84 ms
Reconstruction offset§§	600	600	400
Reconstruction scale§§	550	900	1100
Reconstructed image size (pixels)	1024	1024	1024
Grayscale range	12 bit	12 bit	12 bit
Field of reconstruction||||	50 mm	220 mm	270 mm
Slice thickness	0.25 mm	0.5 mm	1.0 mm
Inter-slice spacing	0.20 mm	0.45 mm	0.9 mm
Cropped image size (pixels)	1,024×1,024	996×265	1,022×149
Number of slices	400	618	422

*420-kV Pantak tungsten X-ray source

^†^RLS Detector: Radiographic Line Scanner (RLS) detector, a 2048-channel gadolinium oxysulfide linear array; this detector, although less sensitive, provides higher in-plane spatial resolution, with a channel pitch of 0.025 mm. It can be used either in a high-resolution mode, with a vertical aperture of 0.25 mm, or (by sacrificing some sensitivity) using a vertical aperture that can vary from 0.5 to 5 mm.

^‡^P250D Detector: Linear array detector consisting of a 512-channel cadmium-tungstate solid-state linear array, which provides superior sensitivity because of its high absorption efficiency. Its vertical aperture (slice thickness) ranges from 5 mm down to 0.25 mm, with a horizontal channel pitch of 0.31 mm.

^§^Material through which X-rays are passed to change beam properties; see Ketcham and Carlson 60 for further explanation.

^||^Medium through which X-ray signal is calibrated; see Ketcham and Carlson 60.

^¶^In offset-mode scanning, center of rotation is laterally offset to increase field of view by percent value listed; see Ketcham and Carlson 60.

^#^Distance between X-ray focal spot and center of rotation.

**Number of rotational positions at which X-rays were collected; see Ketcham and Carlson 60.

^††^Number of consecutive detector channels (‘rays’) averaged during acquisition. For example, a value of 2 for a 2048-channel detector results in 1024 raw data values, and a value of 1 indicates no averaging (2048values). Similar to binning.

^‡‡^Integration time denotes time over which a single detector reading, or sample, is collected, and samples/view denotes the number of samples collected for each view.

^§§^Reconstruction parameters for the CT reconstructor. Offset determines final gray level of wedge material, and increasing scale increases image contrast; see Ketcham and Carlson 60.

^||||^Field of view in final reconstructed image.

**Table 2:** Samples and data outputs.

**Dataset Number**	***Jeholodens***	***Confuciusornis***	‘**Archaeoraptor**’
1.0			
Original Data Format*	16-bit TIF	16-bit TIF	16-bit TIF
Number slices	400	618	422
Individual slice file size	2.049 Mb	518 Kb	330 Kb
Total zipped dataset volume	367 Mb	149 Mb	74 Mb
Voxel dimension X	0.04883 mm	0.2148 mm	0.2637 mm
Voxel dimension Y	0.04883 mm	0.2148 mm	0.2637 mm
Voxel dimension Z	0.20 mm	0.45 mm	0.90 mm
			
2.0			
Resampled cubic voxels	16-bit TIF	16-bit TIF	16-bit TIF
Cubic voxel size	0.04883 mm	0.2148 mm	0.2637 mm
Number slices	1639	1295	1441
Individual slice file size	569 Kb	302–567 Kb	231–401 Kb
Total zipped dataset volume	818 Mb	679 Mb	525 Mb
			
3.0			
Leveled Dataset†	16 bit TIF	16 bit TIF	16-bit TIF
			
3.1			
16bit_XY			
Number slices	400	618	422
Individual slice file size	2.049 Mb	516 Kb	329 Kb
Total zipped dataset volume	365 Mb	159 Mb	73 Mb
			
3.2			
16bit_XZ			
Number slices	1023	996	1024
Individual slice file size	935 Kb	671 Kb	462 Kb
Total zipped dataset volume	846 Mb	585 Mb	421 Mb
			
3.3			
16bit_YZ			
Number slices	277	265	164
Individual slice file size	3.275 Mb	2.520 Mb	2.883 Mb
Total zipped dataset volume	795 Mb	553 Mb	410 Mb
			
4.0			
8bit_leveled	8-bit TIF	8-bit TIF	8-bit TIF
			
4.1			
8bit_XY			
Number slices	400	618	422
Individual slice file size	1.024 Mb	259 Kb	165 Kb
Total zipped dataset volume	114 Mb	64 Mb	30 Mb
			
4.2			
8bit_XZ			
Number slices	1023	996	1024
Individual slice file size	468 Kb	336 Kb	231 Kb
Total zipped dataset volume	201 Mb	144 Mb	93 Mb
			
4.3			
8bit_YZ			
Number slices	277	265	164
Individual slice file size	1.638 Mb	1260 Kb	1.442 Mb
Total zipped dataset volume	179 Mb	126 Mb	89 Mb

*Note: These are ‘unleveled’ and appear as black images when loaded directly into many image viewers; to see imagery adjust image levels in your viewer. Opening the files in Image J automatically sets viewable levels.

^†^These three folders containing leveled 16 bit images viewable in most image viewers; resliced along each orthogonal plane.

**Table 3:** Movies available for download.

**Sample**	**File name**	**File format**	**File size Mb**	**Movie type**
*Confuciusornis*	Conf_XY.MP4	MPEG-4	1.992	Slice stack
	Conf_XZ.MP4	MPEG-4	3.802	Slice stack
	Conf_YZ.MP4	MPEG-4	5.116	Slice stack
	Conf_PitchSpinBodySkel.MP4	MPEG-4	1.082	3-D pitch, matrix removed
	Conf_RollSpinBodyMatrix.MP4	MPEG-4	0.884	3-D roll
	Conf_RollSpinBodySkel.MP4	MPEG-4	1.106	3-D roll, matrix removed
	Conf_RollSpinHeadSkel.MP4	MPEG-4	0.252	3-D roll of skull, matrix removed
	Conf_YawSpinHeadSkel.MP4	MPEG-4	0.249	3-D yaw of skull, matrix removed
	Conf_CONVOID.MP4	MPEG-4	7.609	3-D roll showing voids
‘Archaeoraptor’	Arch_XY.MP4	MPEG-4	1.272	Slice stack
	Arch_XZ.MP4	MPEG-4	3.212	Slice stack
	Arch_YZ.MP4	MPEG-4	4.345	Slice stack
	Arch_SlabSpin.MP4	MPEG-4	1.990	3D slab
				
*Jeholodens*	Jeh_XY.MP4	MPEG-4	5.727	Slice stack
	Jeh_XZ.MP4	MPEG-4	8.764	Slice stack
	Jeh_YZ.MP4	MPEG-4	8.747	Slice stack
	Jeh_RollSpinBodySlab.MP4	MPEG-4	2.476	3-D slab
