# Supplementary material for: X-ray computed tomography datasets for forensic analysis of vertebrate fossils
Source: Sci Data. 2016 Jun 7;3:160040. doi: 10.1038/sdata.2016.40 (PMC4896130; doi:10.1038/sdata.2016.40)
Supplement: Supplementary figures [file sdata201640-s1.pdf]

Supplemental Material for

X-RAY COMPUTED TOMOGRAPHY DATASETS FOR FORENSIC ANALYSIS OF VERTEBRATE FOSSILS

TIMOTHY B. ROWE[1], ZHE-XI LUO[2], RICHARD A. KETCHAM[1], JESSICA A. MAISANO[1], AND MATTHEW W. COLBERT[1]

[1]High-Resolution X-ray CT Facility, Department of Geological Sciences, The University of Texas at Austin, Austin, Texas USA 78712

[2]Department of Anatomy, University of Chicago, Chicago Illinois, USA 60637

Table of Contents

Figure S1 Conventional photograph of the *Confuciusornis* amalgamation………………… 2

Figure S2 Photo of *Confuciusornis* amalgamation with fracture pattern overlay………….. 3

Figure S3 Close-up photo of skull of *Confuciusornis* amalgamation………….………… 4

Figure S4 Close-up of arm of *Confuciusornis* amalgamation……………………………… 5

Figure S5 Close-up of pelvis of *Confuciusornis* amalgamation……………………………. 6

Figure S6 Close-up of leg of the *Confuciusornis* amalgamation…………………….…....... 7

Figure S7 Close-up of feet of the *Confuciusornis* amalgamation………………...……… 8

Figure S8 Volume rendering of *Confuciusornis* amalgamation in top and bottom views…. 9

Figure S9 Key to CT slice positions of the *Confuciusornis* amalgamation………………… 10

Figure S10 Volume rendering of *Confuciusornis* skeleton w/o matrix…………………….. 11

Figure S11 Top surface maps of *Confuciusornis* amalgamation…………………………… 12

Figure S12 Volume rendering of voids in the *Confuciusornis* amalgamation……………..... 13

Figure S13 Key to original CT slices of the Archaeoraptor amalgamation………………… 14

Figure S14 Top surface maps of Archaeoraptor amalgamation…………………………...... 15

Figure S15 Key to original CT slices of *Jeholodens jenkinsi*………………………………. 16

Figure S16 Reconstruction of the skeleton of *Jeholodens jenkinsi*, as preserved…………... 17

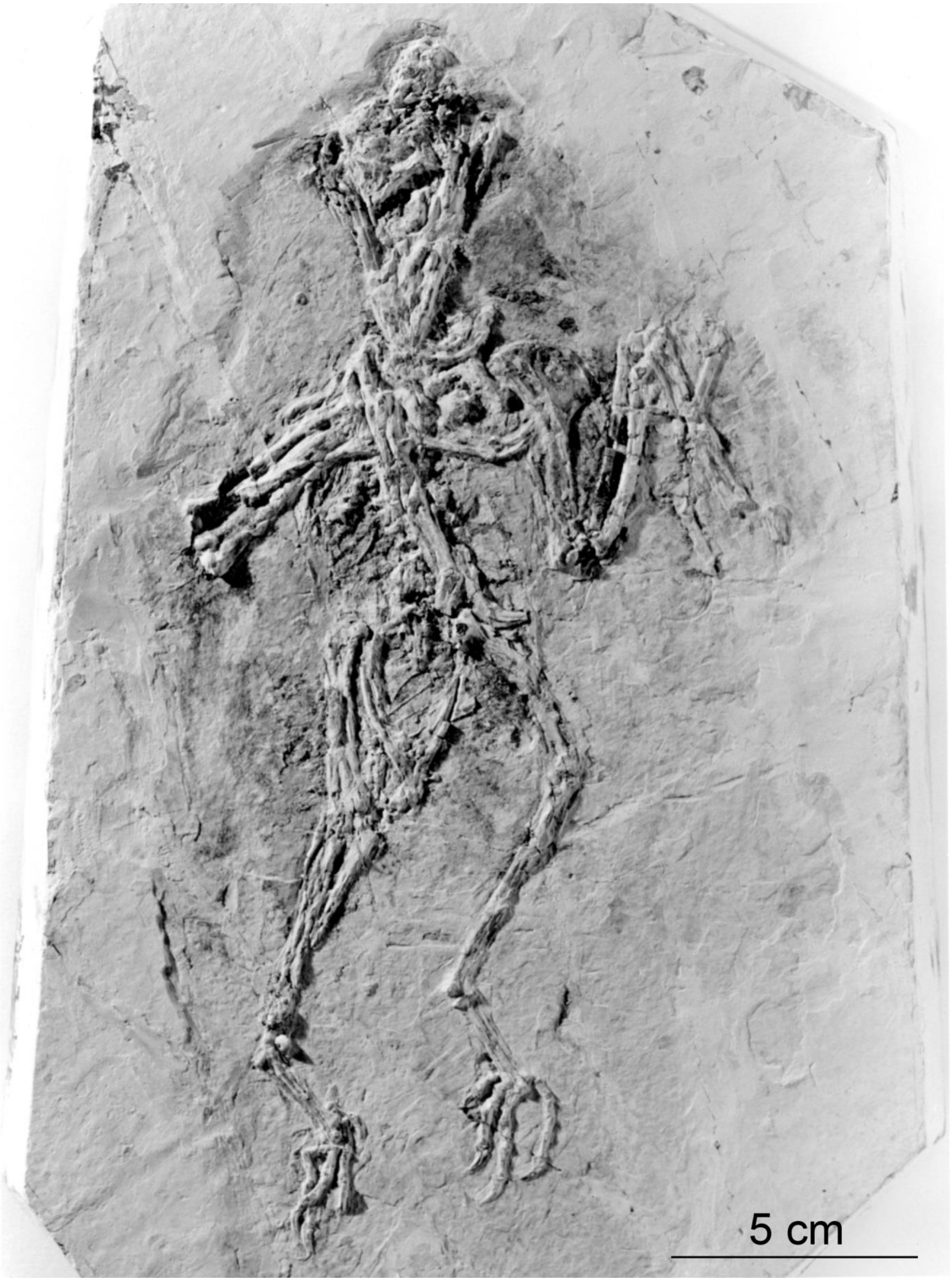

Figure S1. Conventional photograph of the *Confuciusornis amalgamation*, using fine-grained professional black and white film.

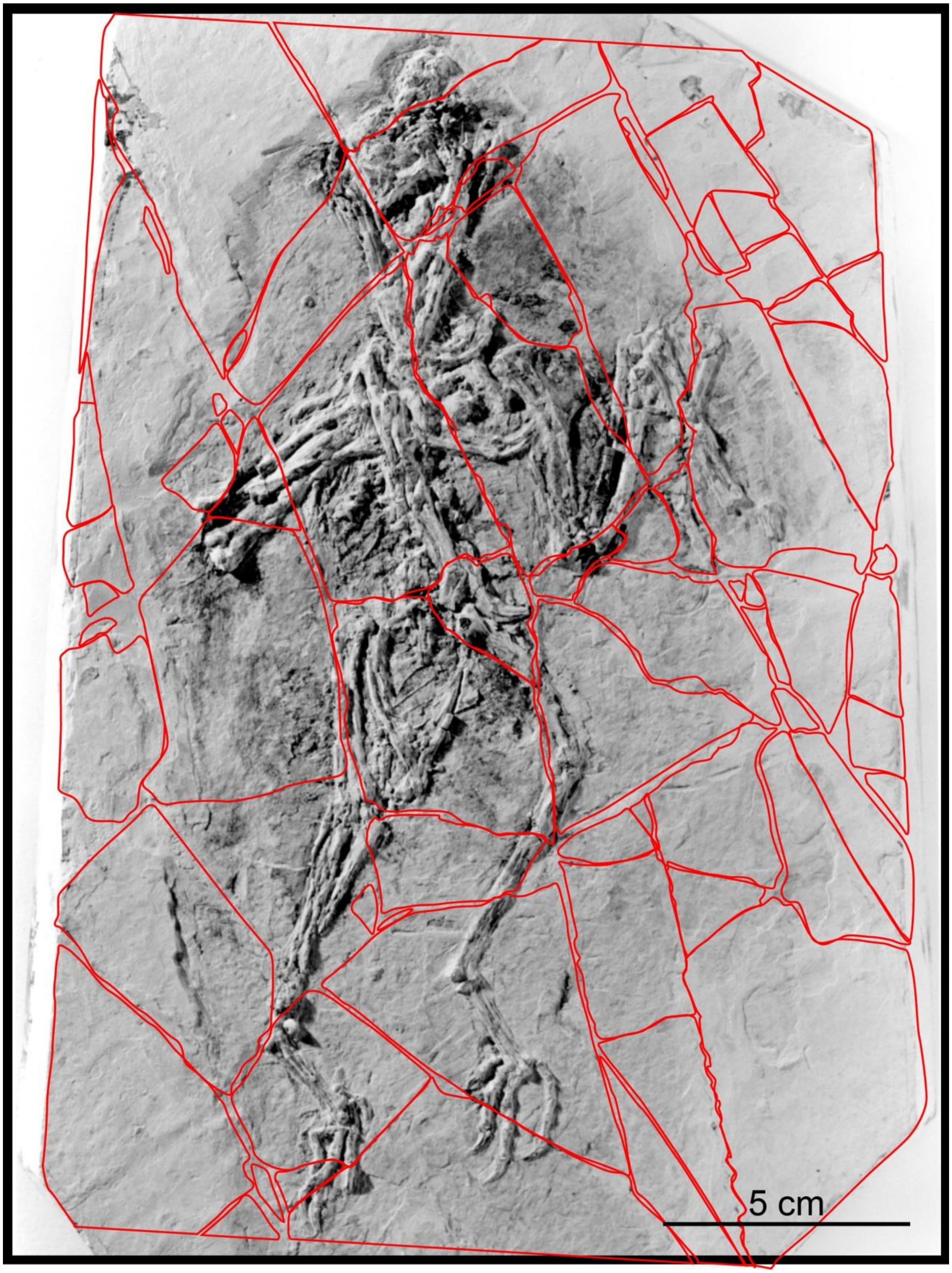

Figure S2. Conventional photograph of the *Confuciusornis amalgamation* with overlay of the fracturing that shattered it before reassembly via mosaicking, and cosmetic treatment of the surface with grout and paint to hide the breakage. The fracture map was generated from the CT dataset, whose axes of orthogonality are shifted slightly from those of the photo (see main Text)

Figure S3. Close-up photograph of the skull of the Confuciusornis amalgamation.

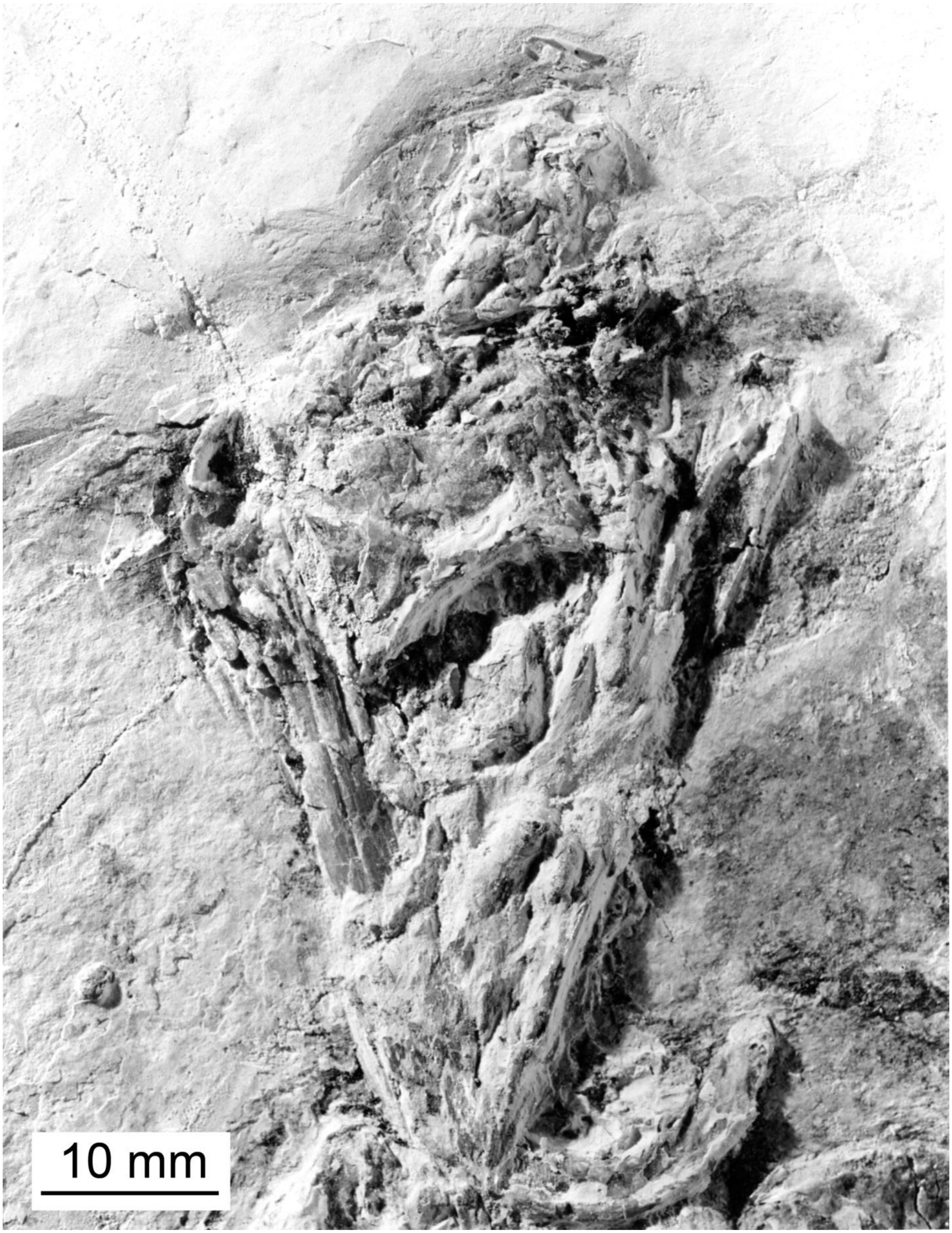

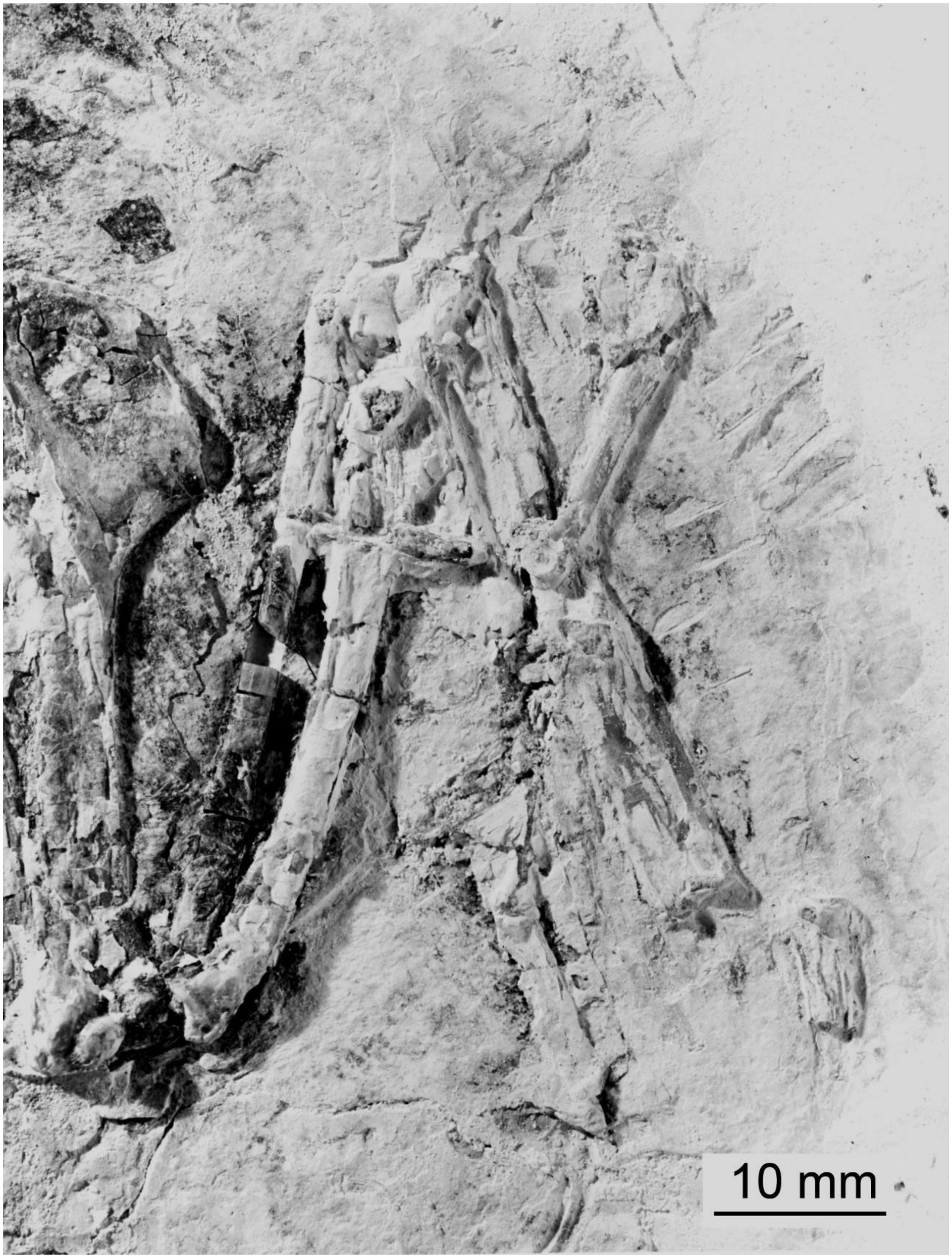

Figure S4. Close-up photograph of the left arm and hand of the *Confuciusornis* amalgamation.

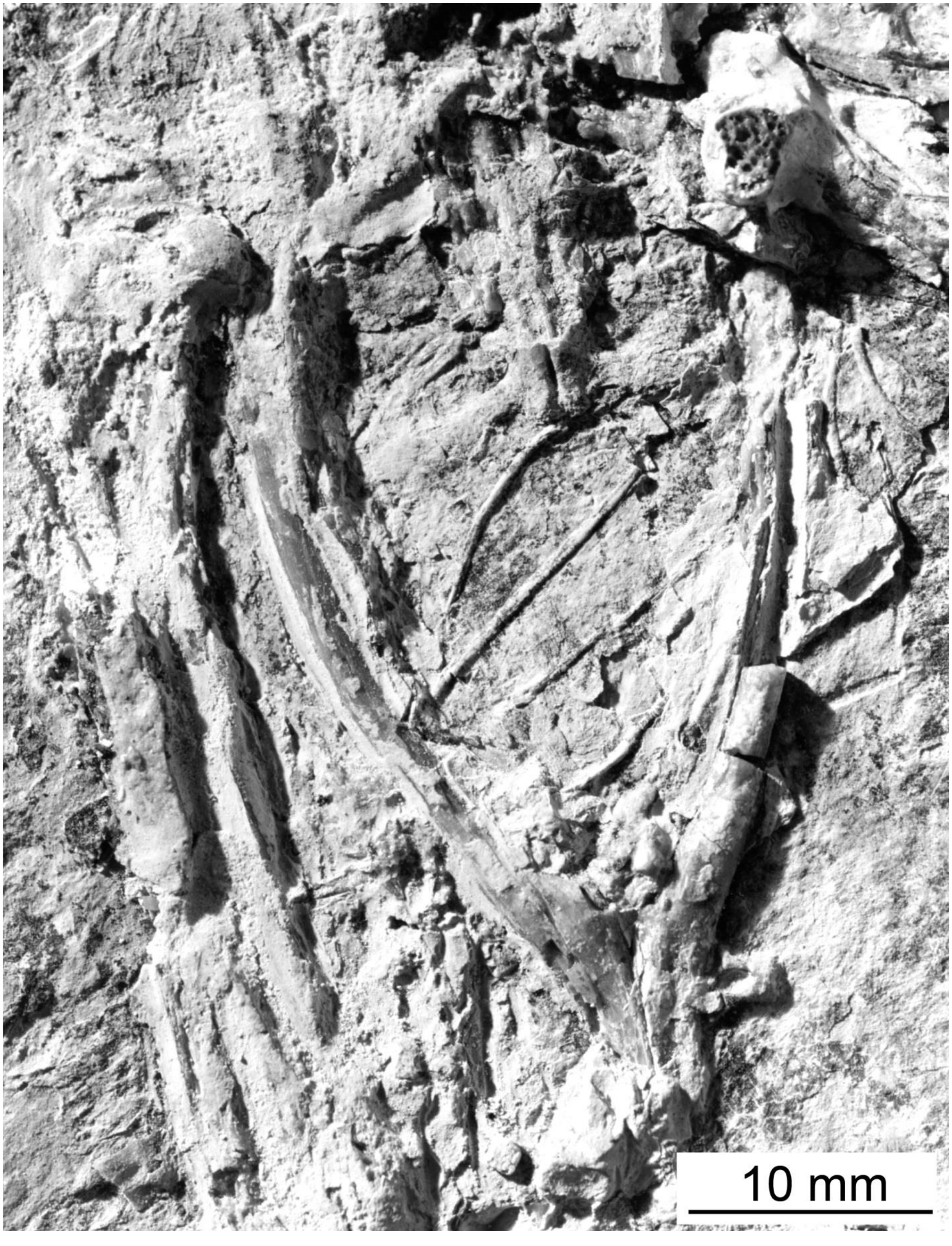

Figure S5. Close-up photograph of the pelvis and right femur of the *Confuciusornis* amalgamation.

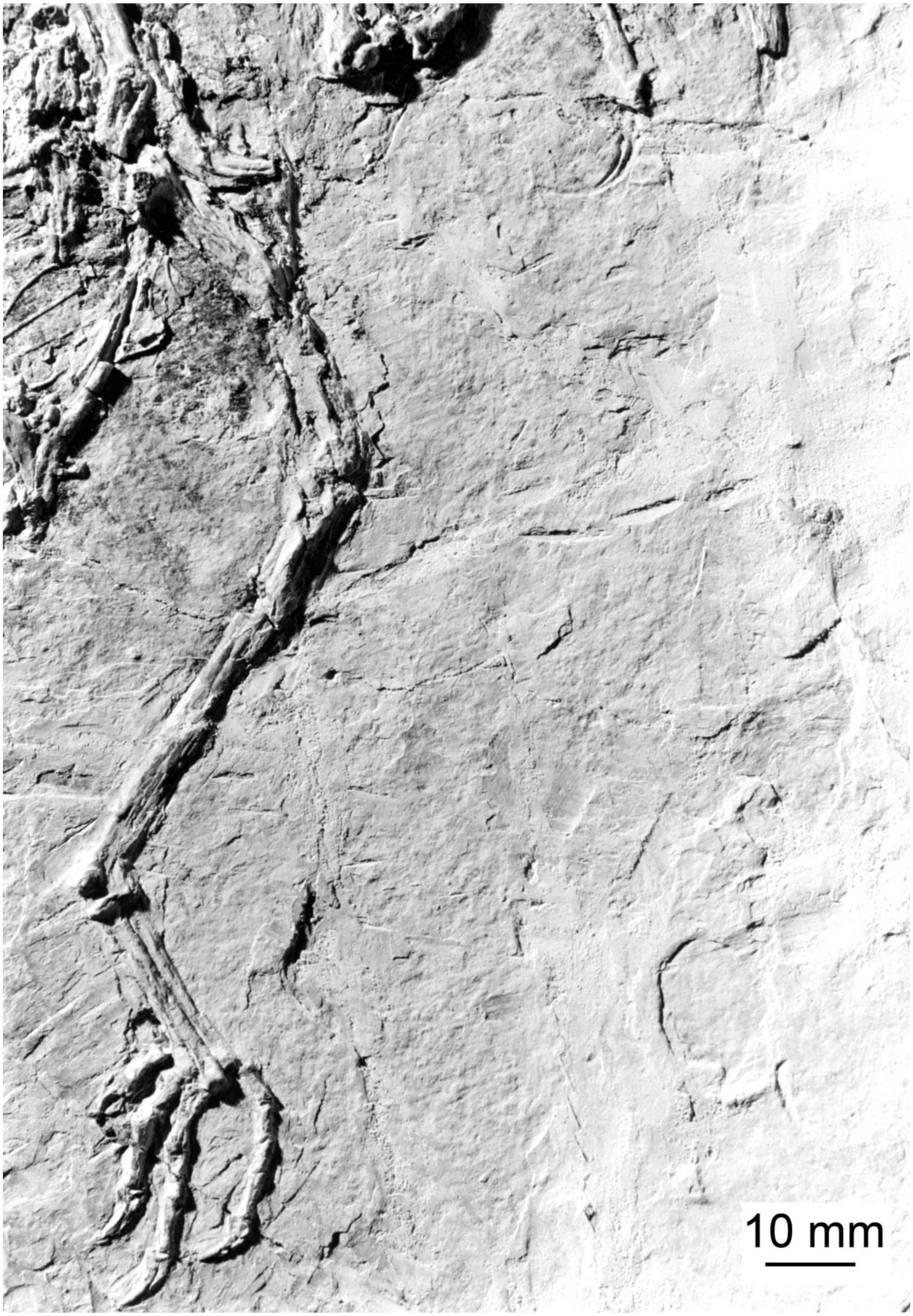

Figure S6. Close-up of the left leg of the *Confuciusornis* amalgamation.

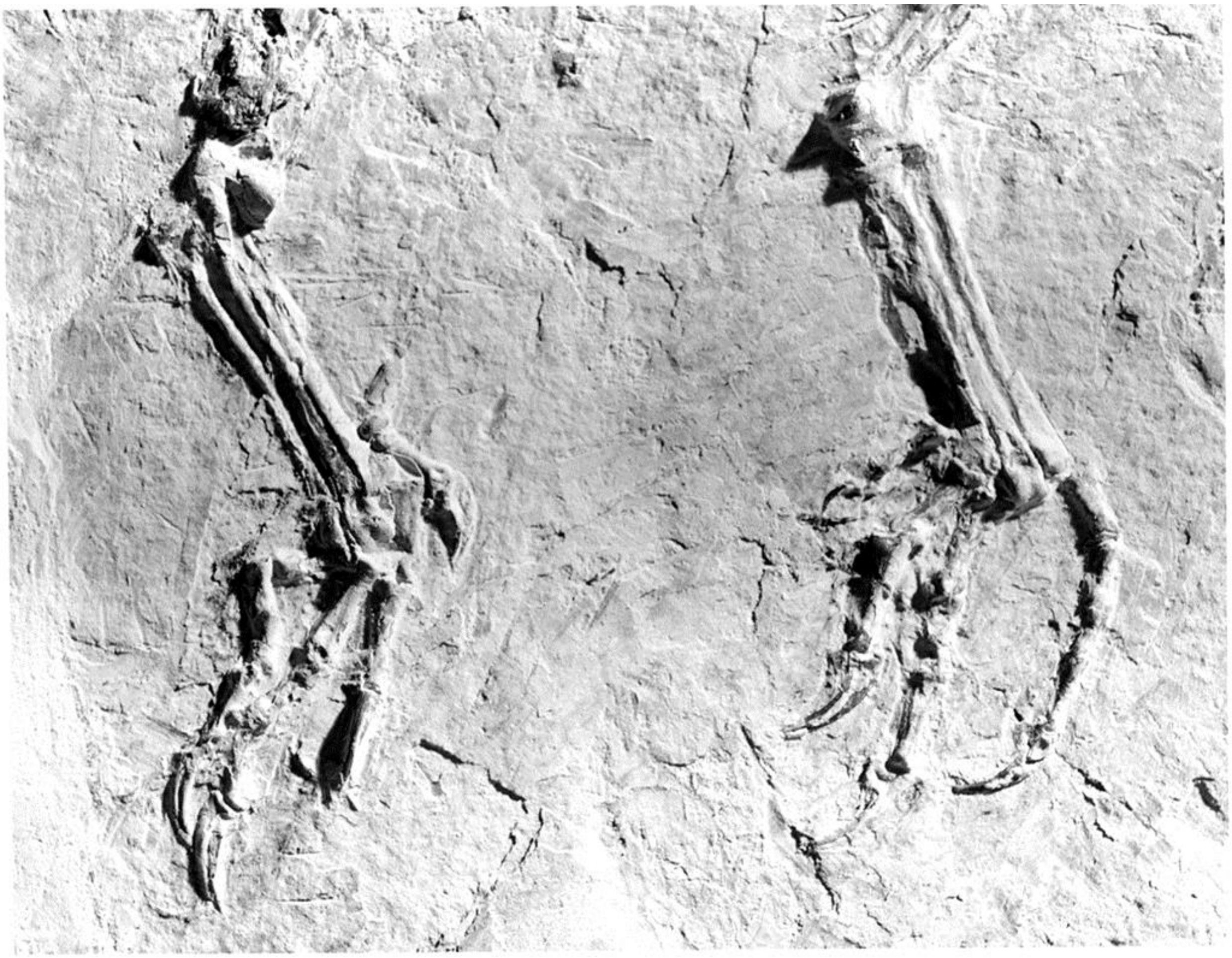

Figure S7. Close up photo of feet of *Confuciusornis* amalgamation

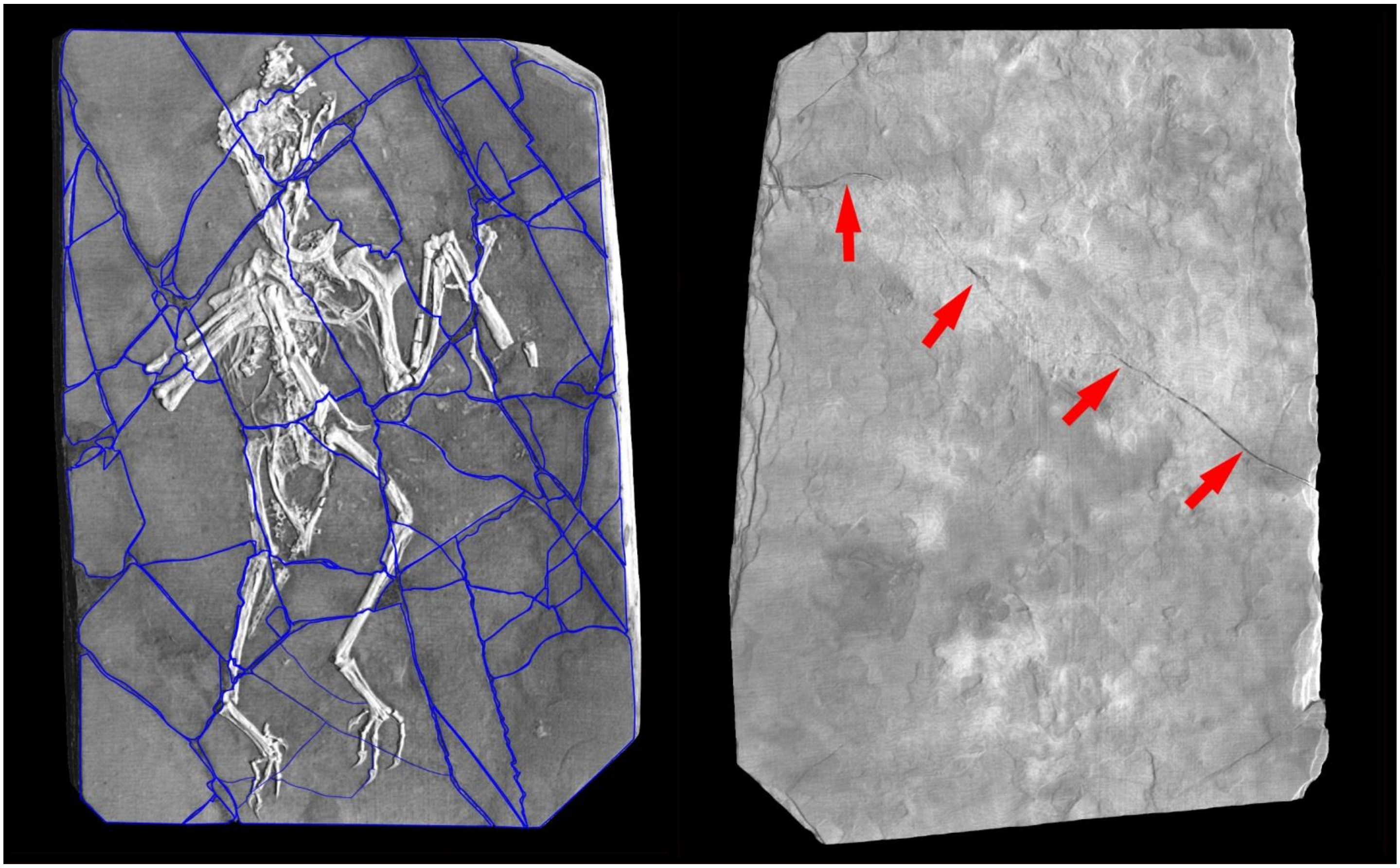

Figure S8 Volume rendering of the *Confuciusornis* amalgamation in top and bottom views, with fracture pattern of top layer mapped in blue, and the very different fracture of the backing slab (red arrows). Mis-matched fracture patterns on either side of an object such as this is symptomatic of an amalgamation. Generated from CT dataset (Data Citation 1) using *VGStudioMax 2.1* (Data Citation 1).

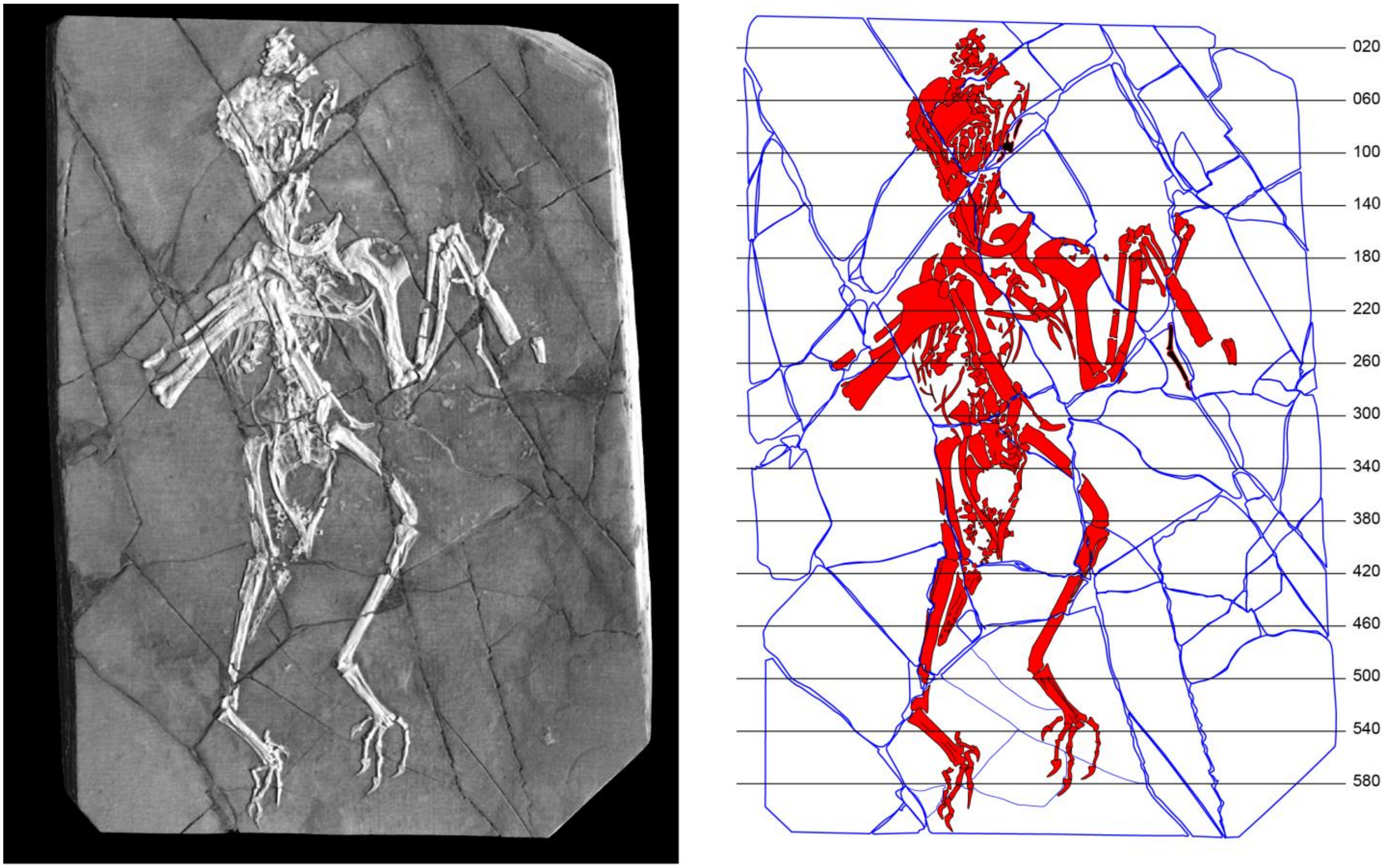

Figure S9. Key to original CT slices of the *Confuciusornis* amalgamation. Volumetric reconstruction of the *Confuciusornis* amalgamation (left), and a map of the skeleton (red) and shatter-fracture pattern in blue of the surface layer (right), keyed to slice numbers in XY plane (Data Citation 1).

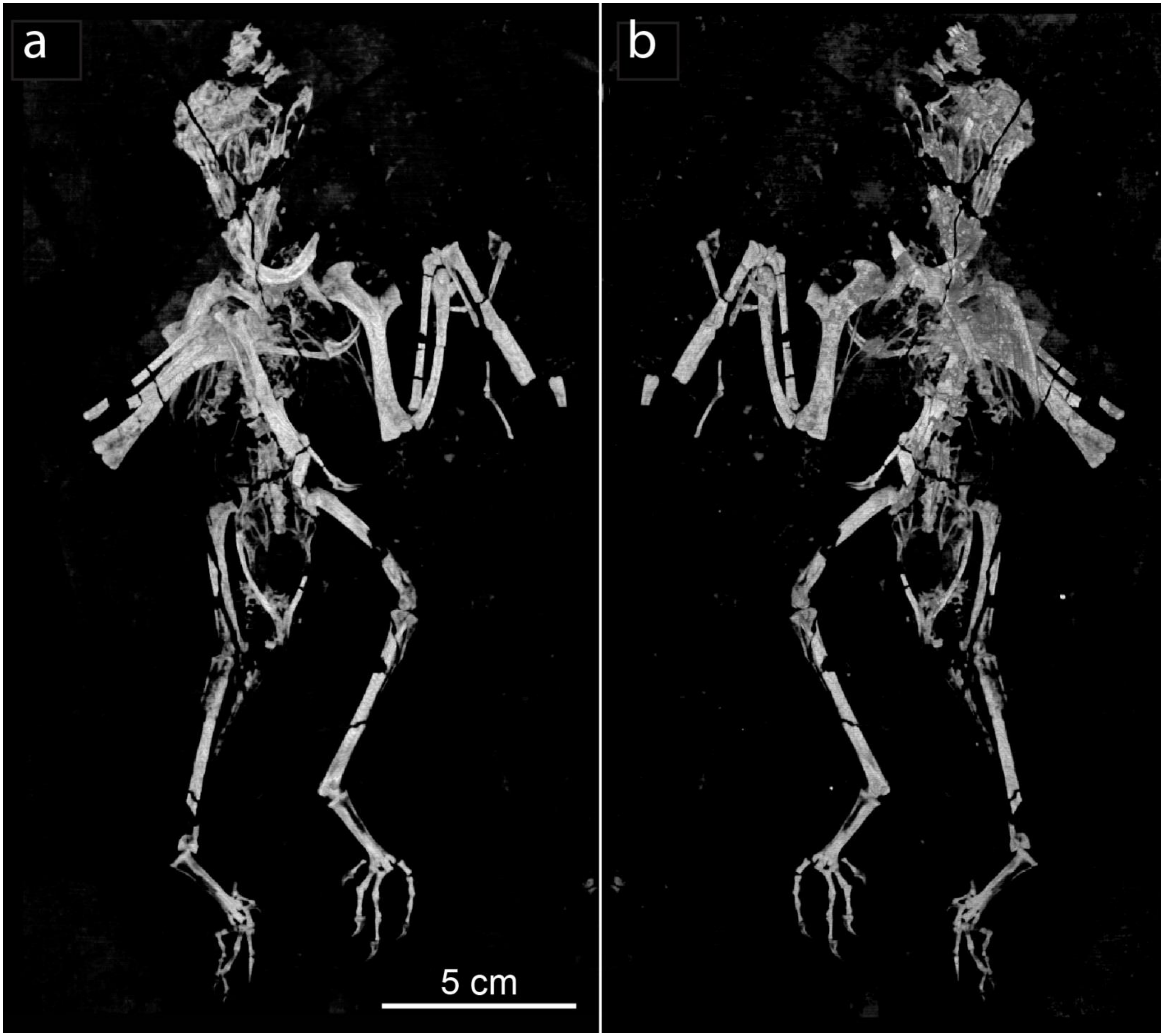

Figure S10 Volume rendering of the *Confuciusornis* skeleton in dorsal (a) and ventral (b) views, with matrix filtered as transparant; generated from CT dataset (Data Citation 1) using *VGStudioMax 2.1* (Conf_PitchSpinBodySkel.mov, Conf_RollSpinBodySkel.mov, Data Citation 1).

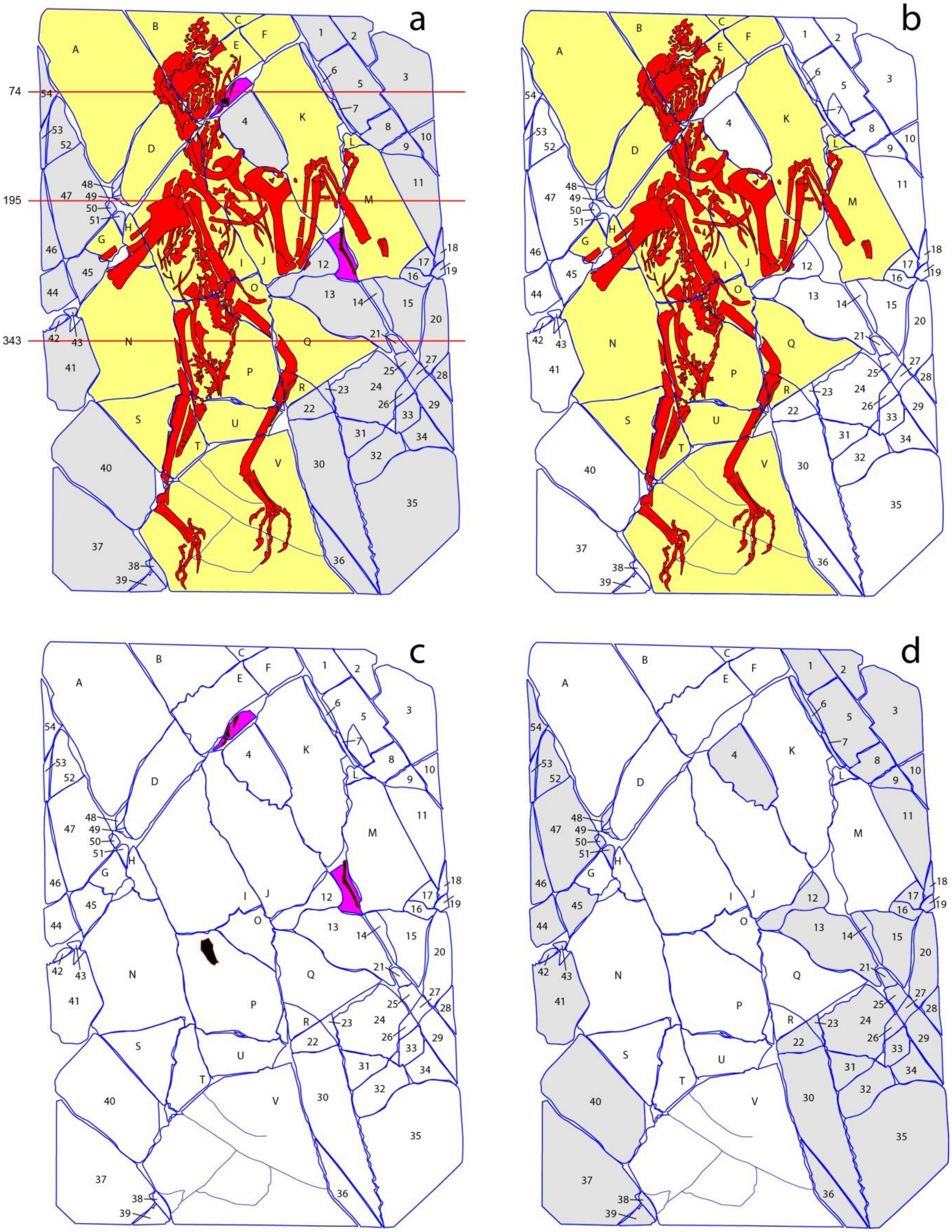

Figure S11. The complete top surface (a); verified bone-bearing pieces (b); extraneous bone-bearing pieces (c), and barren shims (d).

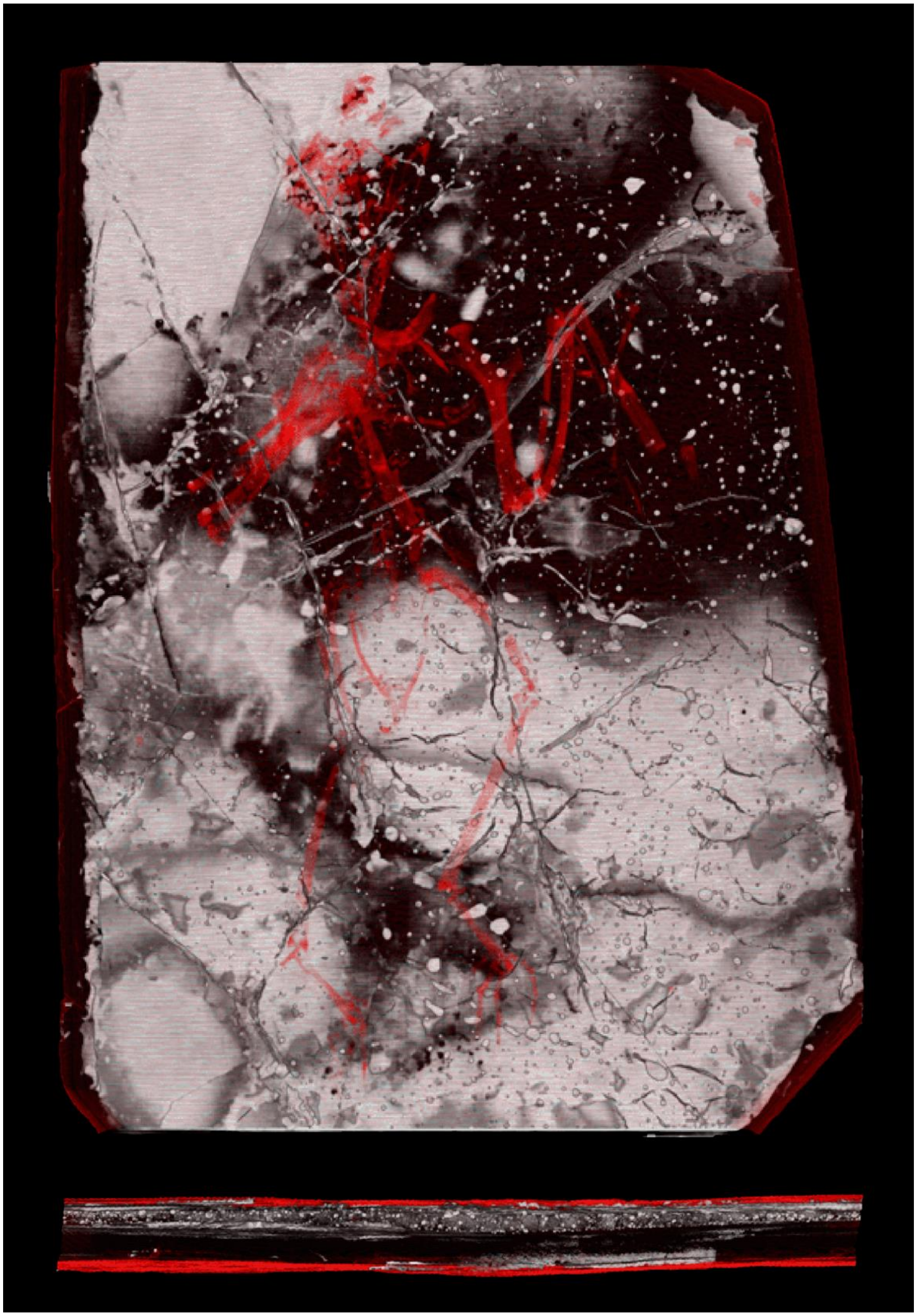

Figure S12. Volume rendering of voids in the *Confuciusornis* amalgamation in grayscale, with solid elements rendered translucent (black), and the surface of the skeleton and whole block in red, viewed from the top (above) and the edge (below). In edge view, note the concentration of spherical bubbles in the grout, and delamination (black) of both top and back slabs (CONVOID.mov; Data Citation 1).

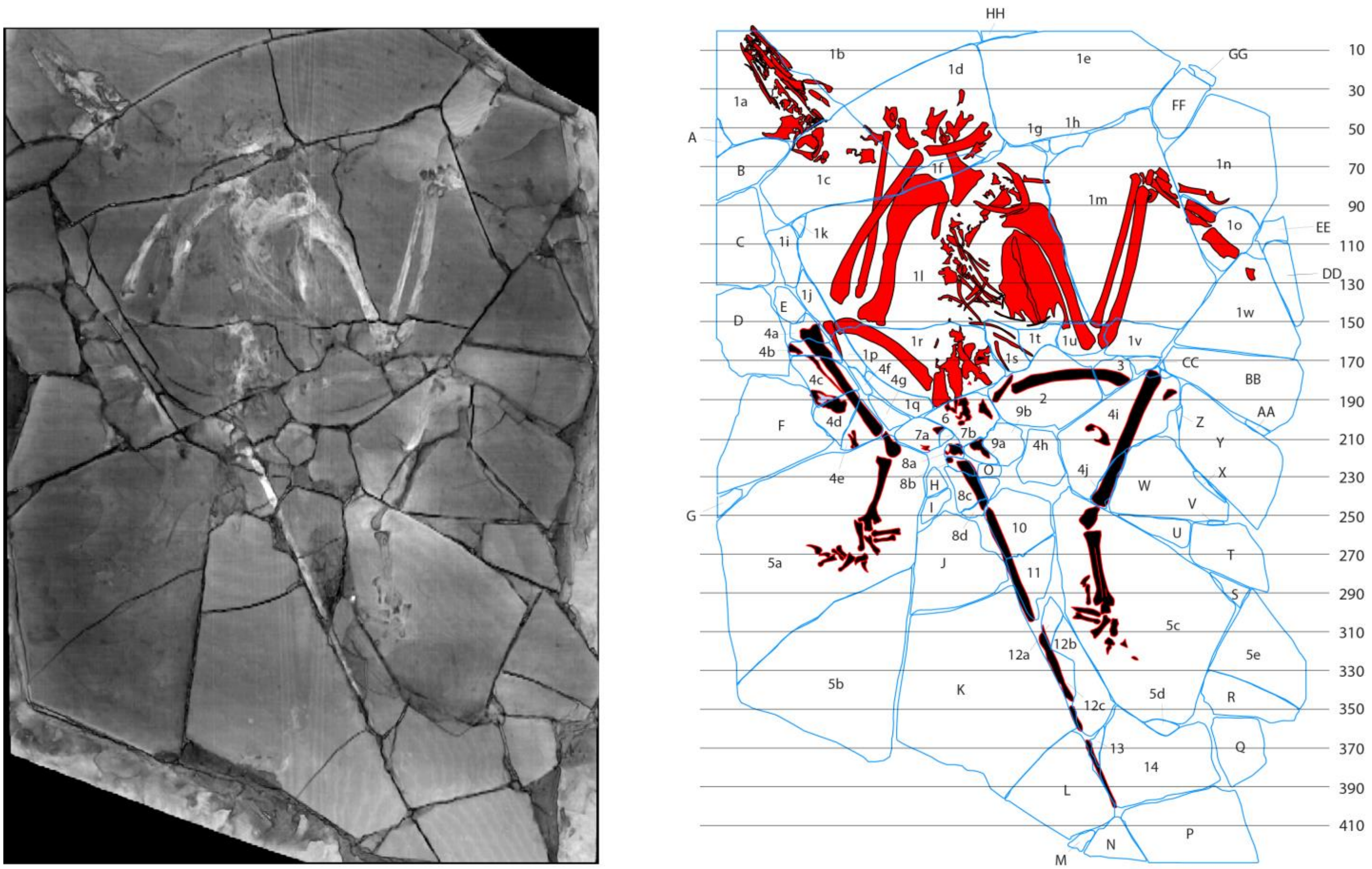

Figure S13. Key to original CT slices of the *Archaeoraptor* amalgamation. Volumetric reconstruction of the *Archaeoraptor* amalgamation (left), and a map (right)of the bird skeleton (red), and the extraneous elements (black), and shatter-fracture pattern in blue of the surface layer, keyed to slice numbers in XY plane.

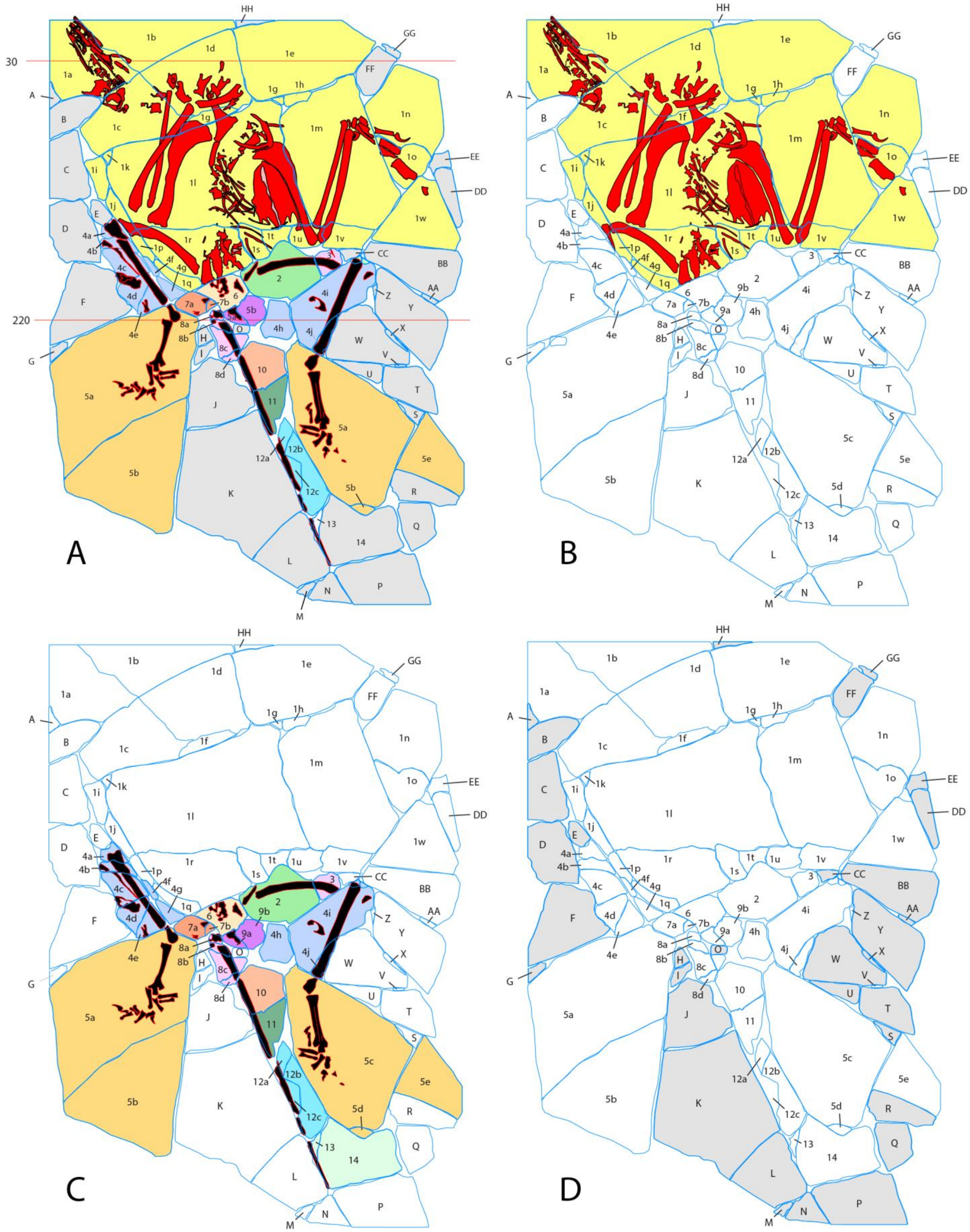

Figure S14. Archaeoraptor amalgamation: The complete top surface (a); verified bone-bearing pieces (b); extraneous bone-bearing pieces (c), and barren shims (d).

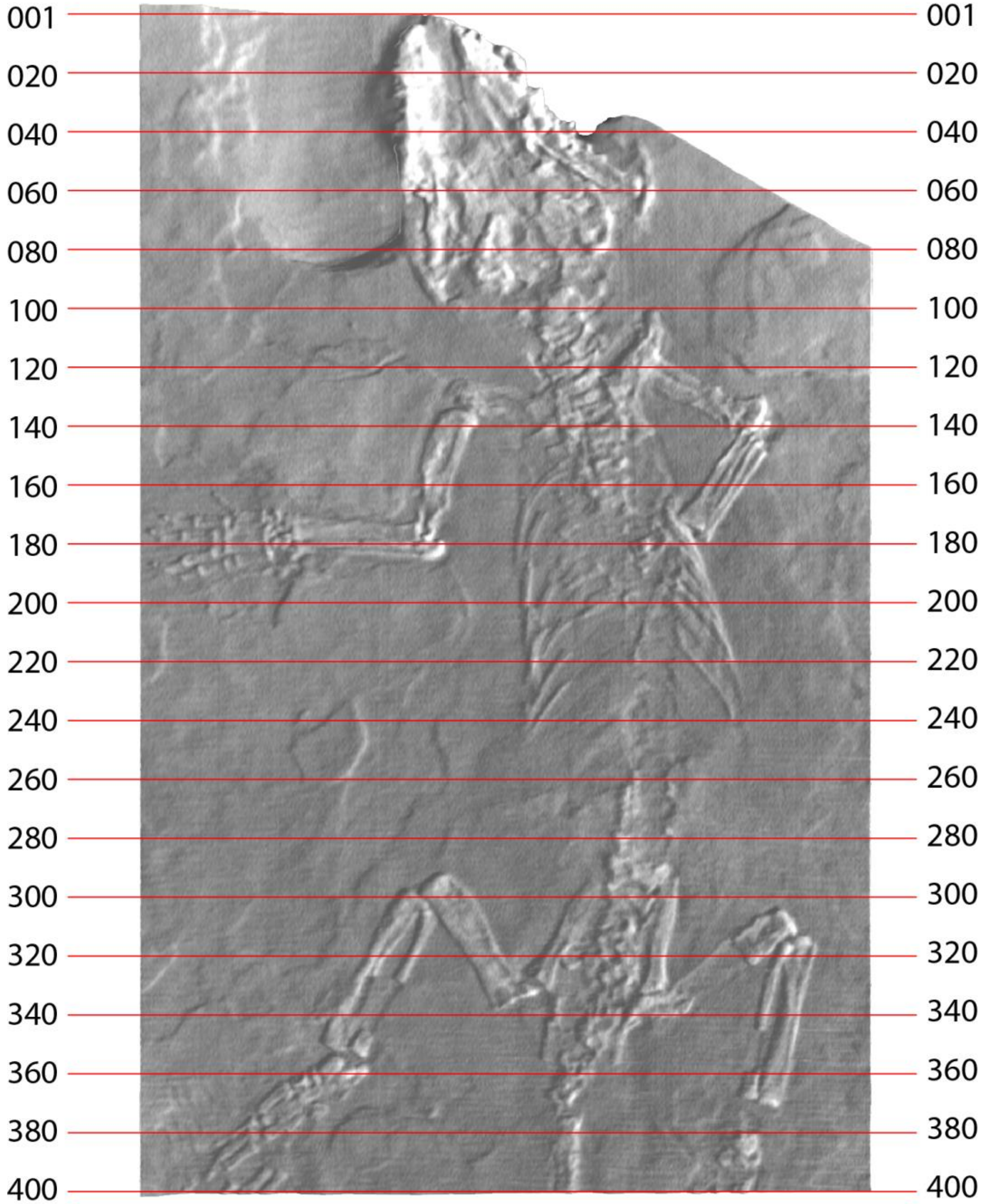

Figure S15. Key to positions of the original CT slices of *Jeholodens jenkinsi*..

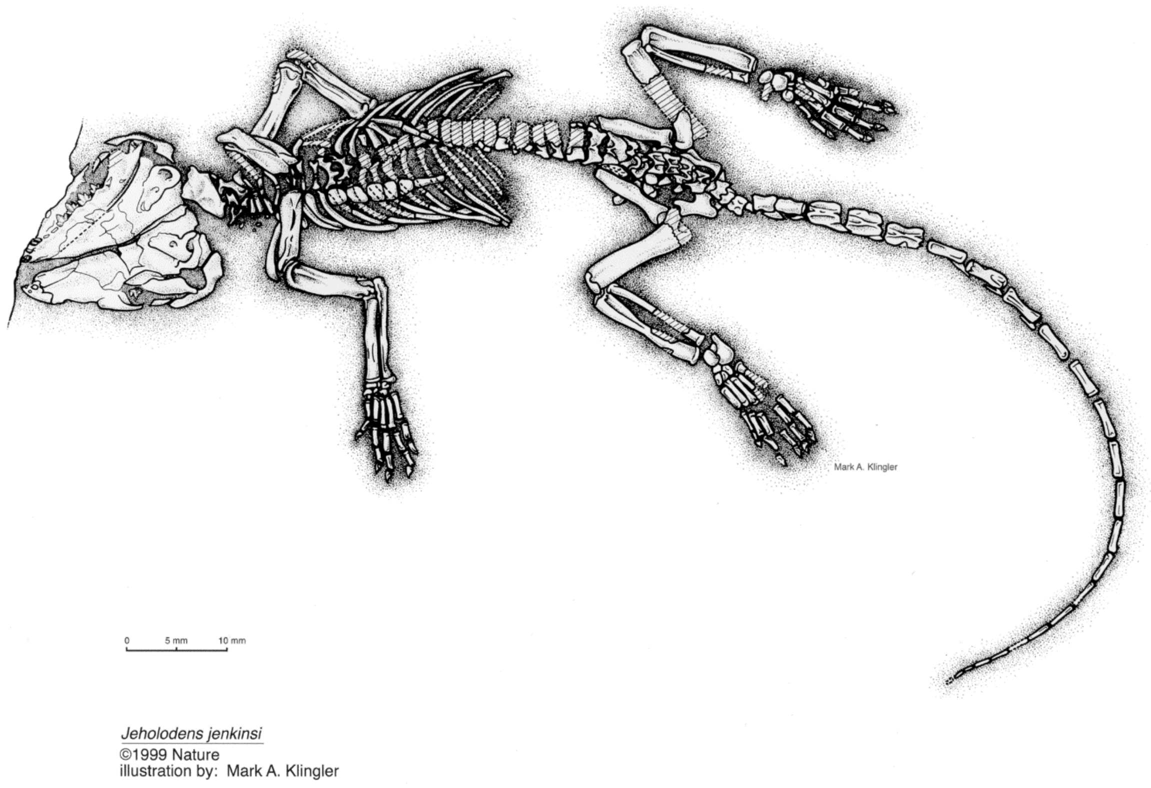

Figure S16 Reconstruction of the skeleton of *Jeholodens jenkinsi*, as preserved.
